# The climate cost of lateness: decomposing historical drivers of CO_2_ industrial emissions during Spanish industrialization and deindustrialization, 1890–2021

**DOI:** 10.1007/s44498-026-00077-1

**Published:** 2026-04-20

**Authors:** Ángel Sanjuán-Ruiz, Juan Infante-Amate, Eduardo Aguilera

**Affiliations:** 1 Department of Economics and Economic History, University of Seville, Seville, Spain; 2 Department of Economic Theory and History, University of Granada, Granada, Spain; 3 Institute of Economics, Geography and Demography, Spanish National Research Council (CSIC), Madrid, Spain

**Keywords:** Industrial ecology, CO_2_ emissions, Decomposition analysis, Sectoral approach, Time series, Spain

## Abstract

Industry accounts for ~ 34% of global CO₂ emissions, positioning it as the largest single contributor to anthropogenic climate change. Understanding the historical trajectory is essential for effective mitigation, yet most analyses are short-term and long-term studies rarely examine industry. This article reconstructs consistent long-run series of industrial CO₂ emissions in Spain from 1890 to 2021, disaggregated by source (direct, indirect, and process-related) and by sector. Combining newly harmonized historical energy accounts with decomposition techniques (IPAT and additive LMDI), we quantify the relative contribution of value added, energy intensity of production, carbon intensity of energy use, and structural change to industrial CO_2_ emissions trajectories. Emissions increased twenty-sevenfold between 1890 and 2007, with the sharpest rise occurring during 1950–1973. Spain’s path reflects a double delay: late industrialization produced a compressed, fossil-fuel-intensive take-off, while delayed adoption of efficient and low-carbon technologies after the 1970s reinforced carbon lock-in. Improvements in energy and carbon intensity have been consistently outpaced by output growth, revealing rebound effects and the limits of relative decoupling. As a late joiner, Spain shows how delayed industrial development shaped CO₂ emissions trajectories, as the dominance of non-metallic and building-materials industries produced a volatile, non-linear decline in carbon intensity, keeping it above early-industrializer levels into the twenty-first century. Deep decarbonization therefore requires targeted interventions in a small set of structural “hotspots,” alongside technological upgrading and material circularity to overcome the historical legacy of late development.

**Supplementary Information:**

The online version contains supplementary material available at 10.1007/s44498-026-00077-1.

## Introduction

Contemporary societies face a global environmental challenge deeply rooted in historical processes. The intensive use of fossil fuels, beginning during first industrialization, has led to a substantial accumulation of greenhouse gases (GHGs) in the atmosphere and a steady rise in global average temperatures (IPCC, 2022). In 2018, the industrial sector accounted for 24% of direct global emissions, rising to about 35% when including emissions from electricity consumption (Lamb et al., 2021).

Today, China alone accounts for 24% of global industrial emissions, despite significant technological advances (Liu et al., 2021; Wang & Feng, 2018). In contrast, European industries have achieved significant reductions through improvements in both energy and carbon intensity, facilitated by technological change and energy transitions over the long term (Kander et al., 2013, 2020), as well as slower growth in output (Crippa et al., 2023). Nonetheless, regional disparities persist, particularly between Northern European countries such as Germany and the UK, and Southern economies like Italy and Spain (Bhattacharyya & Matsumura, 2010).

This trajectory diverges sharply from that of the early industrial period. During the nineteenth century, Western Europe and North America accounted for the bulk of global fossil-fuel CO₂ emissions, largely as a result of economic and demographic expansion (Henriques & Borowiecki, 2017; Lindmark, 2004; Malanima, 2024). Meanwhile, organic-based Asian economies underwent a process of deindustrialization (Pomeranz, 2000). However, the rise of Asia in the global economy from the 1970s onward (Baldwin, 2018) brought about a major geographical shift in the center of gravity of global emissions (Andrew & Peters, 2023). In parallel, the twentieth century also witnessed the emergence of late-joiners, such as Spain, which followed a different pattern of industrial development—characterized by rapid and compressed growth facilitated by the adoption of pre-existing technologies (Gerschenkron, 1962).

Achieving meaningful reductions in global emissions without undermining economic growth requires substantial gains in carbon intensity—that is, lowering emissions per unit of output (Infante-Amate et al., 2024; Riahi et al., 2017; Vogel & Hickel, 2023). These gains can be realized through technological and structural transformations that reduce energy intensity (i.e., energy per unit of output) (Grübler et al., 2018; Rogelj et al., 2015; Slameršak et al., 2022), or lower the carbon intensity of energy consumption (i.e., emissions per unit of energy) (Anderson & Peters, 2016; Budinis et al., 2018; Liu et al., 2021; Minx et al., 2018).

These interlinked dynamics are commonly examined using Kaya decomposition analysis (Kaya, 1989). Numerous studies have shown that improvements in carbon intensity have been insufficient to offset the impact of economic growth on global emissions (Feng et al., 2015; Guan et al., 2008; Hubacek et al., 2021; Lamb et al., 2021). In both the EU and China, industrial output growth has been the dominant driver of emissions, while gains in carbon intensity—mainly through energy efficiency—have played a lesser role (Feng et al., 2018; Liu et al., 2015; Kopidou et al., 2016; Shao et al., 2016). Structural change has also had only limited short-term influence (Feng et al., 2018; Kopidou, 2016). Moreover, CO₂ reductions remain particularly difficult in a handful of energy-intensive subsectors responsible for most emissions (Matsumoto et al., 2019; Wen et al., 2022).

In Spain, CO₂ emissions rose gradually during the first half of the twentieth century and accelerated sharply between 1950 and 1975 due to late industrialization and oil dependency. Since 1980, emissions have followed a volatile path (Esteve & Tamarit, 2012; Infante & Aguilera, 2024). High carbon intensity—partly rooted in elevated energy intensity and structural inefficiencies—has persisted relative to similar economies (Bartoletto & Rubio, 2008; Moutinho et al., 2017). The growing relevance of non-metallic minerals, particularly during the real estate boom, further offset intensity gains in the early twenty-first century (Cansino et al., 2015; Tarancón & del Río, 2007).

In industry, energy intensity gains have been insufficient to offset direct emissions boosted by growing production since 1995, where a small group of carbon-intensive sectors—cement, iron and steel, and chemicals—have consistently dominated emissions (Cansino et al., 2015; Mountinho et al., 2017; Tarancón & del Río, 2007). However, most existing studies focus on the post-1995 period, use aggregated data, and often omit indirect and process-related emissions, which account for the 43% and 19% of global industrial CO₂ (Rissman et al., 2020).

This study addresses that gap by reconstructing CO₂ emissions by process, source, and sector for the period 1890–2021 in Spain—the first to offer this level of disaggregation extending back to the nineteenth century for any country. The choice of Spain as a case study rests both on the availability of historical data that make this unprecedented reconstruction possible and on the specificity of its industrialization trajectory. Early industrialization (1890–1930s) was constrained by limited availability of coal (Coll & Sudrià, 1987; Sudrià, 1995). Electrification facilitated broader development in the 1920s (Betrán, 2005), but progress was soon disrupted by the Civil War and subsequent Francoist autarky. The 1950s liberalization reforms unleashed rapid industrial growth driven by cheap oil imports (Carreras & Tafunell, 2018). This transition marked Spain’s entry into an industrial metabolic regime (Infante-Amate et al., 2015) during the late industrialization phase (1950–1980). A second surge in energy and material use occurred in the 1990s, spurred by quarrying activity and a real estate boom (Alcántara & Padilla, 2021; Infante et al., 2021) even as the economic structure became increasingly deindustrialized.

Within this historical context, the objectives of this study are threefold:

(i) To trace the trends in industrial CO₂ emissions in Spain by process, source and sector covering the industrial history of the country;
(ii) To identify the main drivers behind emissions trajectories;
(iii) To examine intersectoral and temporal differences in emissions intensity, especifically in key emission hotspots.

## Methods

### Boundaries, sources and estimation

In this study, we provide a consistent long-run disaggregation of industrial CO₂ emissions into seven sectors from 1890 to 2021, which—due to sources availability—we further expand to 32 subsectors from 1960 onward (see Supplementary Information SI1 – Sect. 3.1). In our accounting, we distinguish between three emission sources: direct emissions from the combustion of fossil fuels; indirect emissions from electricity generation; and process emissions from material production where CO₂ is released independently of energy consumption (see Supplementary Information SI1 – Sect. 1).

The absence of harmonized long-term series on energy consumption and sectoral emissions prior to 1990—common across most countries—is largely due to the methodological fragmentation of national accounting systems and the frequent reclassification of industrial sectors. Therefore, the main challenge of this paper has been to carry out an extensive process of compilation, correction, and harmonization of historical industrial statistics (for more detailed information, see Supplementary Information SI1 – Sect. 2).

For the period 1990–2021, we used physical energy consumption data from IDAE (2024). For the earlier period, 1958–1990, we relied on two official archival sources: the *Estadística Industrial de España* (1958–1977) and the *Encuesta Industrial* (1978–1992). These sources contain varying classification systems and inconsistent units of energy measurement, necessitating manual revision and standardization of all data series. The *Anuario Estadístico de España* provides additional data on coal consumption (1948–1956) and electricity use in electro-intensive industries (1937–1957). However, systematic records for other energy sources are largely absent prior to 1958, prompting a dual methodological strategy.

On one hand, we drew from secondary literature to reconstruct final consumption of coal (Coll & Sudrià, 1987), biomass (Infante-Amate et al., 2022), hydropower (Nadal, 1992) and electricity (Bartolomé, 2007) for seven benchmark years: 1890, 1925, 1932, 1933, 1934, 1948 and 1951. On the other hand, we applied a bottom-up approach to disaggregate these estimates using sector-specific energy intensity observations (GJ/ton) collected from different primary and secondary sources (see Supplementary Information SI1 – Table 3).

Final energy consumption was converted into primary energy using appropriate conversion factors. In the case of electricity, the annual power generation mix was considered (Aguilera et al., 2019). Carbon emissions were estimated using standard emission factors for both direct and indirect emissions. In addition, process-related emissions were extrapolated based on the Inventario Nacional de Emisiones (2024), moving backwards from 1990 according to the physical trends in the production of key materials (see Supplementary Information SI1 – Table 5).

For the period between 1890 and 1958, carbon intensities were estimated for seven benchmark years for which information is available, using industrial value-added data from Prados de la Escosura (2017). These estimates were applied to annual production Fig.s to reconstruct a continuous long-term series of industrial CO₂ emissions.

### Decoupling and decomposition analysis

We analyze the drivers of industrial CO₂ emissions (C) using an additive LMDI decomposition method (Ang, 2005, see Supplementary Information SI1 – Sect. 4.1). In our model, variations in C are explained by changes in four key factors:

i. Industrial activity (A), measured by value added at constant 2010 euros.
ii. Energy intensity (e), defined as gigajoules of final energy consumed per 2010 euros of value added.
iii. Carbon intensity of energy use (m), expressed in kilograms of CO₂ per gigajoule of final energy consumed.
iv. Structural change (s), captured by the share of each industrial branch in total value added (sᵢ = Aᵢ/A). This component reflects the impact of shifts in the relative weight of different branches on total emissions.

Where:

- ${s}_{i}=\frac{{A}_{i}}{A}$, refers to the share of sector *i* in total value added (productive structure).
- ${e}_{i}=\frac{{E}_{i}}{{A}_{i}}$, refers to the energy intensity of sector *i*.
- ${m}_{i}=\frac{{C}_{i}}{{E}_{i}}$, refers to the carbon intensity of sector *i*.

Then:

$$C=\sum_{i}A\cdot {s}_{i}\cdot {e}_{i}\cdot {m}_{i}$$

In this study, we also apply a model inspired by Tapio (2005) to examine the degree of decoupling between industrial value added and CO₂ emissions (see Supplementary Information SI1 – Sect. 4.2). We identify five possible scenarios: (i) Dirty growth: emissions grow at a faster rate than value added; (ii) Weak decoupling: value added grows faster than emissions; (iii) Strong decoupling: value added increases while emissions decline; (iv) Recessive mitigation: emissions decline more rapidly than value added; (v) Dirty recession: value added declines more rapidly than emissions.^1^

Additionally, we assess which mitigation strategy has played a more significant role: the reduction in energy intensity or in the carbon intensity of energy consumption. In this regard, we identify four possible outcomes: (i) Dual efficiency: both variables decrease; (ii) Dual inefficiency: both variables increase; (iii) Energy intensity efficiency: only energy intensity decreases; (iv) Energy mix efficiency: only carbon intensity decreases.

## Results

### Historical trends in industrial CO_2_ emissions in Spain

**Fig. 1:**
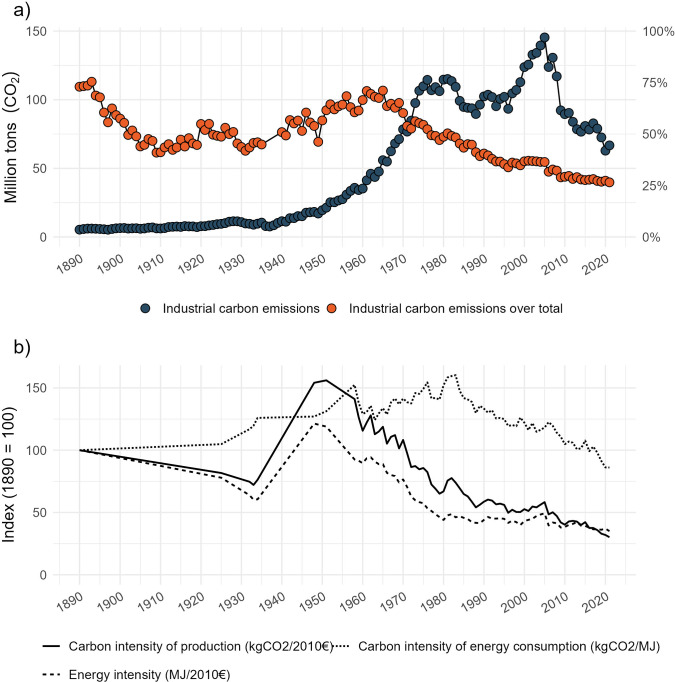
Evolution of CO_2_ industrial emissions in the Spanish manufacturing and emissions as a share of total CO_2_ emissions in Spanish economy (**a**); carbon intensity of production, energy intensity and carbon intensity of energy use in industry (**b**). Total emissions data in panel (**a**) are sourced from Andrew and Peters (2023) for fossil fuels and cement, and estimatid in this study for other industrial processes. For the period 1890–1958, the series in panel (**b**) are constructed from seven benchmark years and linearly interpolated between them, which smooths short-term fluctuations. From 1958 onward, annual data are available, accounting for the higher year-to-year volatility visible in the later part of the series. The data for this Figure is available in Zenodo: https://zenodo.org/records/17900036

Industrial CO₂ emissions in Spain increased twenty-sevenfold between 1890 and 2005. After peaking in 2005, they declined by 54% through 2021. Their share over total economy decreased, moving from 73 to 26% over the analyzed period (Fig. 1a). From 1890 to 1925, emissions grew at an average annual rate of 1.7%. Meanwhile, the share of industrial emissions in total emissions declined, largely due to the coal-fueled expansion of the railway system during the early stages of industrialization. Carbon intensity of production also decreased—that is, CO₂ emissions grew at a slower pace than value added (Fig. 1b). During the Francoist autarky (1939-1950s). industry became the main emitter, exceeding 50% of total emissions by 1942, as carbon intensity rose due to increasing energy intensity. From 1950 onward, emissions grew at an accelerated rate, averaging 7.9% annually between 1959 and 1974, as Spain completed its industrialization process. In 1965, the share of industrial emissions in total emissions peaked at 71%.

From the mid-1970s onward, the trend of carbon emissions began to show a non-linear evolution (Fig. 1a), shaped by structural transformations, economic crises, and the major decrease in carbon intensity (Fig. 1b). Within this context, industrial emissions over total experienced a sustained decline until 1996 (Fig. 1a). Between 1999 and 2007, emissions accelerated once again, reaching a peak in 2005 with 145 Mt of CO₂ emitted. After the 2007 crisis, emissions experienced the sharpest decline—by 48.9% until 2021.

The carbon intensity of industrial production has been influenced by changes in its two core components: the final energy intensity of production (i.e., the energy use per output) and the carbon intensity of energy use (i.e., the CO_2_ emissions per energy consumed, explained by the energy mix). While both carbon intensity of production and energy intensity followed an N-shaped trajectory, the carbon intensity of energy followed an inverted U-shaped trend over the long term. Between 1890 and 1934, the fall in production carbon intensity was mainly driven by lower energy intensity (Fig. 1b). In the 1940s, this pattern reversed, peaking in the early 1950s. Carbon intensity then resumed its decline—first due to falling energy intensity (c.1950–1980), and later to improvements in the carbon intensity of energy use from the 1980s onward.

**Fig. 2:**
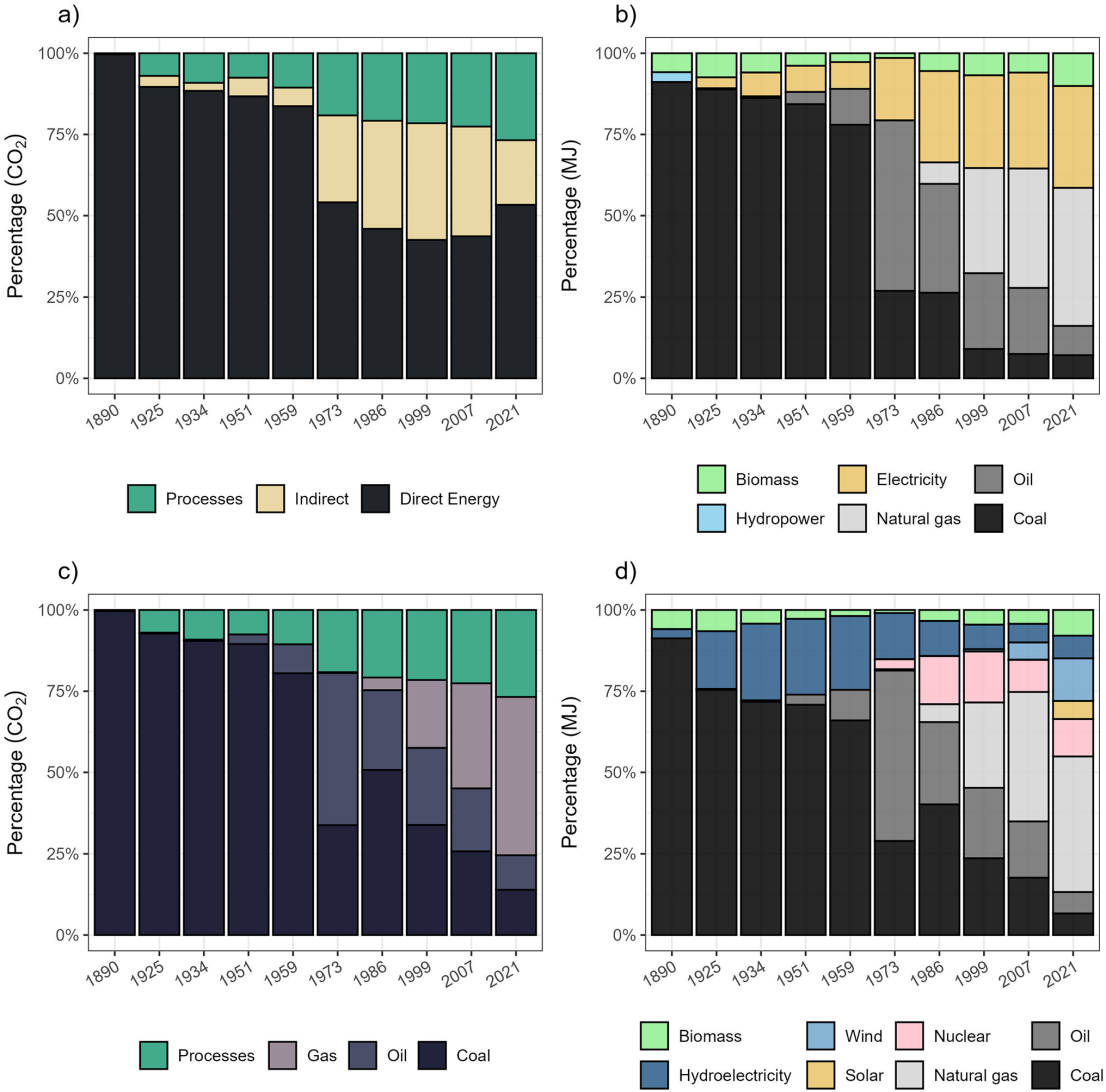
Composition of CO_2_ emissions (kgCO_2_/2010€) by process (**a**); Final energy mix in industry (**b**); Composition of CO_2_ emissions (kgCO_2_/2010€) by source (**c**); Primary energy mix in industry (**d**). The data for this Figure is available in Zenodo: https://zenodo.org/records/17900036

Changes in carbon intensity are closely linked to various processes of technological change and energy transition. First, they are associated with the energy quality of the energy carriers finally consumed. Figure 2a shows the predominance of direct emissions until the mid-twentieth century. From 1959 onward, however, indirect emissions and those from industrial processes gained greater relevance, accounting for 36% and 22%, respectively, by 1999. The rise in indirect emissions is directly related to the growing share of electricity in the final energy mix, as shown in Fig. 2b, which enabled a more technologically efficient use of energy. A comparison of Figs. 1b and 2b reveals that periods of rising electricity consumption correspond to phases of substantial improvement in energy intensity (1890–1934 and 1951–1986), whereas its relative stagnation or decline coincided with periods of increasing or stagnant energy intensity (1934–1951 and 1986–2007).

Second, carbon intensity was shaped by successive transitions toward primary energy sources with lower carbon content. Coal-related emissions dominated until the 1950s, after which the energy transition toward oil during the 1960s reshaped the emissions composition. Following the oil shocks, there was a partial return to coal between 1979 and the early 1990s, followed in turn by a transition toward natural gas (Fig. 2c). Lastly, the growing consumption of carbon–neutral electricity (Fig. 2d) has contributed to reducing the carbon intensity of energy and explains the slight decline in the share of indirect emissions after 2007 (Fig. 2a).

**Fig. 3:**
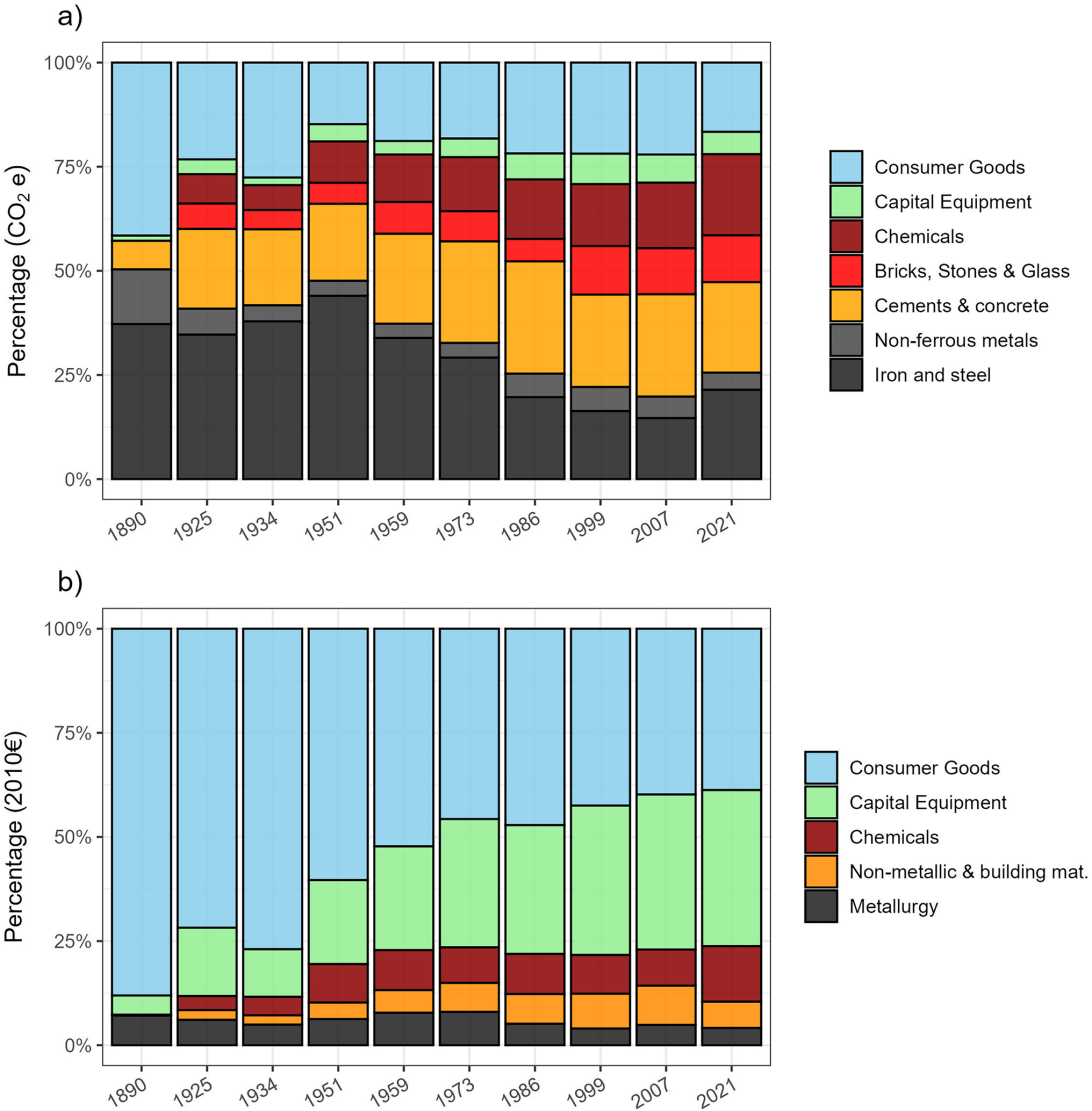
Sectoral composition of CO_2_ emissions in Spanish industry (**a**) and of value added in constant prices (2010€) (**b**). Value added data in panel (**b**) are sourced from Prados de la Escosura (2015). The data for this Figure is available in Zenodo: https://zenodo.org/records/17900036

Changes in the industrial structure also influenced trends in carbon intensity, as the expansion of more carbon-intensive activities affects the overall system's carbon intensity. In 1890, consumer goods industries accounted for 41% of CO2 emissions (Fig. 3a), but 88% of industrial value added (Fig. 3b). In contrast, heavy and high-carbon-intensive sectors—specifically building materials, chemicals, and metallurgy—were responsible for 57% of emissions while generating just 7% of value added. By 1951, this inverse relationship had deepened: industrial output became increasingly dominated by Capital Equipment and, to a lesser extent, by these high-emission sectors, which accounted for 81% of total industrial emissions.

From 1959 onward, Capital Equipment industries gained overall prominence within the industrial structure, while building materials experienced relative growth between 1999 and 2007. The share of CO₂ emissions became increasingly concentrated in carbon-intensive activities: the combined emissions accounted for 78% of total industrial CO₂ emissions in 1959—and, despite internal fluctuations, still represented 78% by 2021 (Fig. 3a).

**Fig. 4:**
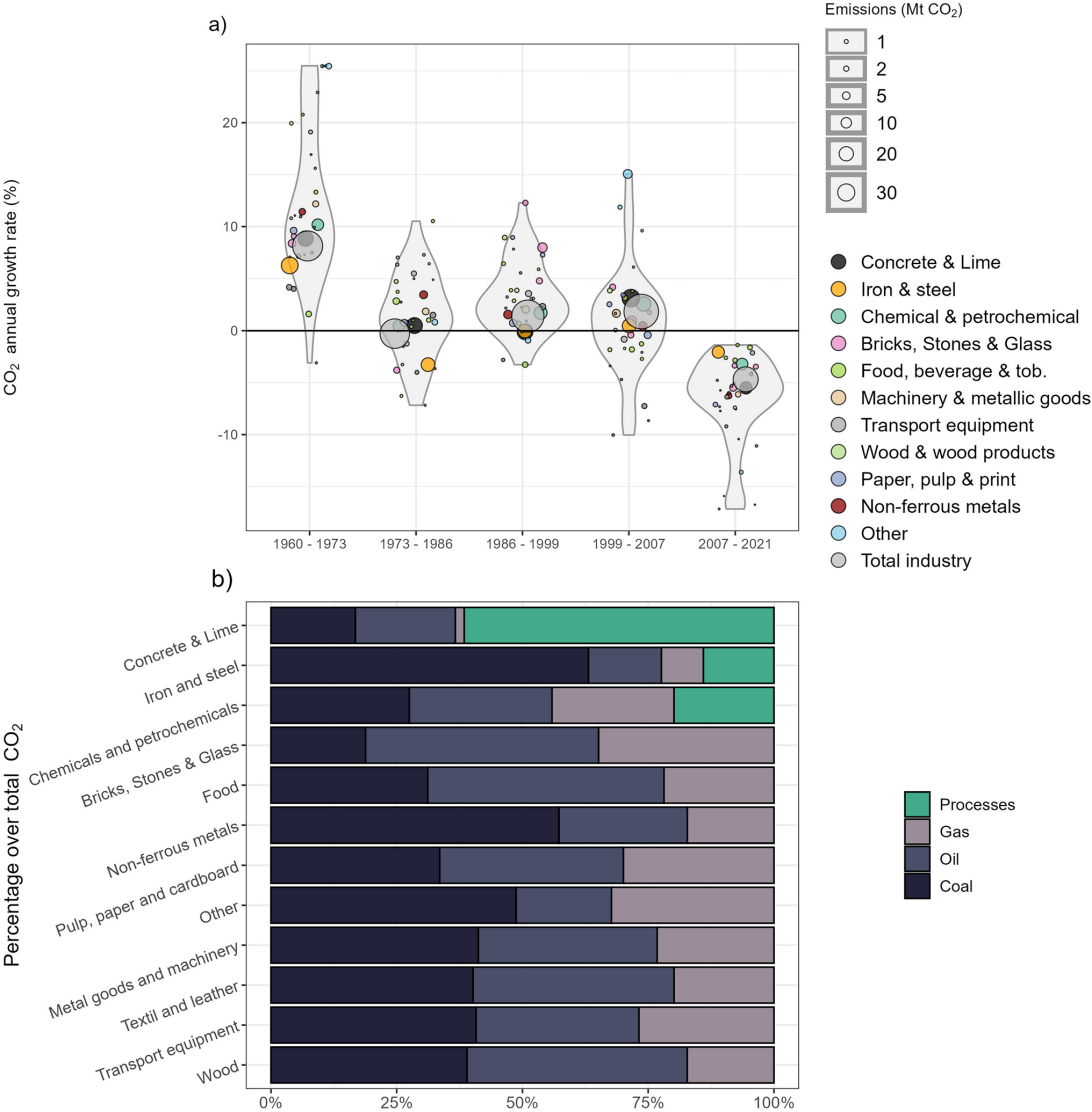
Annual growth rate of CO_2_ emissions by sector. The size represents the volume of emissions per sector (**a**). Cumulative carbon emissions by sector and source (**b**). Sub-sectoral level information is provided in Supplementary Information SI1 – Figure S2 and F S3. The data for this Figure is available in Zenodo https://zenodo.org/records/17900036

Due to their central role within the industrial structure and their technical characteristics, a small number of activities have concentrated and shaped overall emissions trends (Fig. 4a). During both the first acceleration phase (1960–1973) and the second (1999–2007), these key sectors expanded at a pace closely aligned with that of total industrial output. Furthermore, these activities exhibited emission profiles that diverged significantly from the rest of the industrial system, reflecting technical constraints to emissions reduction.

While oil dominated until 1980, coal until 1990, and natural gas thereafter, this overall pattern masks notable distinctions among the largest emitters (Fig. 4b). For example, process-related CO₂ emissions accounted for 61.5% in the cement industry and 20% in chemicals. In the iron and steel industry, coal-derived emissions—primarily from coke combustion—accounted for 63.1% of total emissions, due to coke’s dual role as both a thermal energy source and a chemical reducing agent in blast furnace operations. These characteristics imply that reducing carbon emissions cannot rely solely on lowering energy consumption, as a substantial portion of emissions originates from non-energy processes or industrial uses unrelated to energy conversion.

### Decoupling CO_2_ emissions from industrial production

**Fig. 5:**
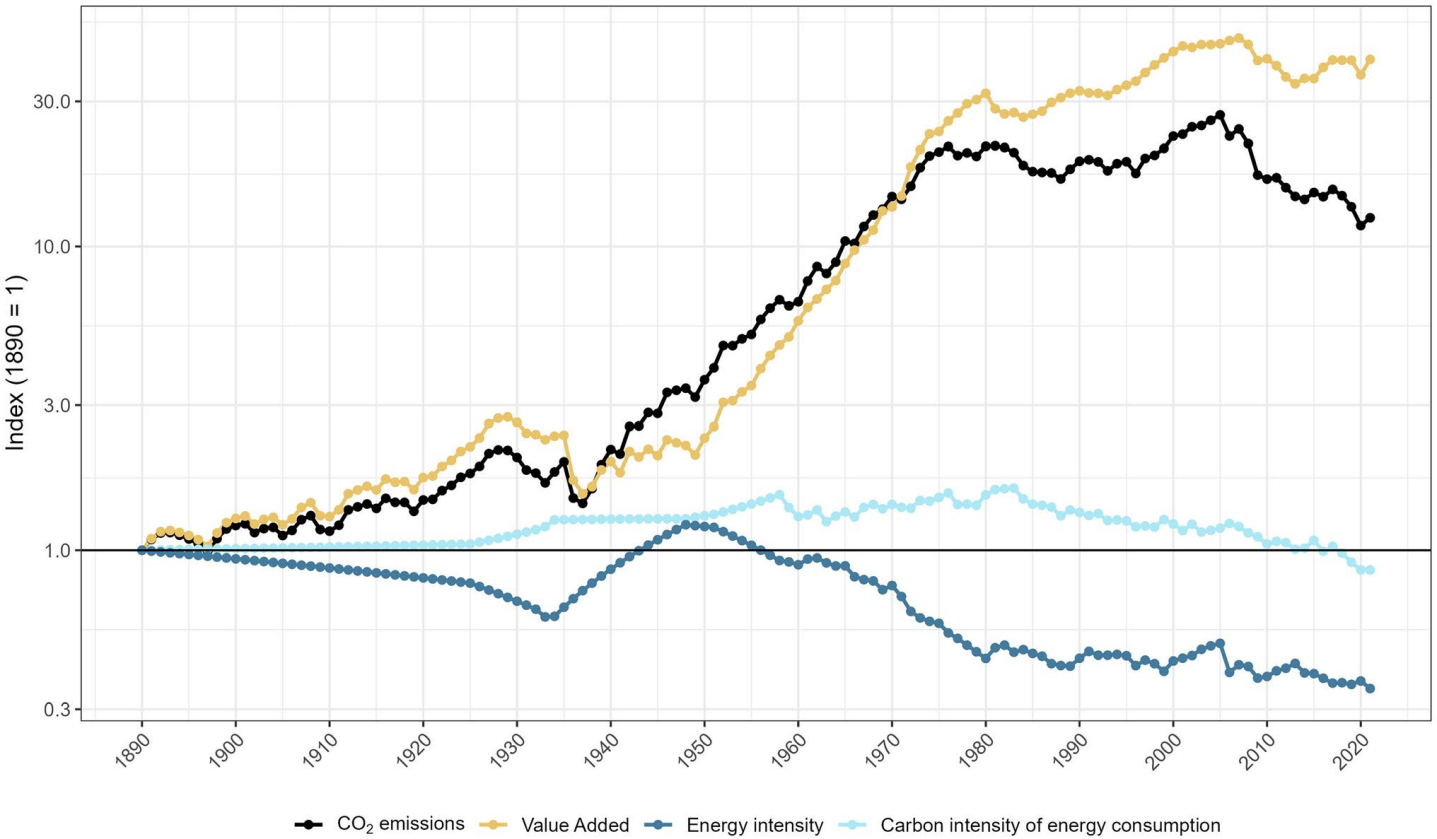
Trends of CO_2_ emission drivers across the entire industry. Values are indexed where 1890 = 1. The data for this Figure is available in Zenodo https://zenodo.org/records/17900036

Figure 5 presents the long-term trajectories of the variables included in the IPAT model. Up to 1934, industrial output and CO₂ emissions rose at nearly the same pace, in a period marked by modest gains in energy intensity and a slight increase in the carbon intensity of energy. Between 1934 and 1948, industrial CO₂ emissions began to rise more rapidly than industrial output, resulting in a growing divergence reversed in the 1950s, when production began to grow at a faster rate than emissions. In other words, a relative—or weak—decoupling emerged between industrial production and CO₂ emissions since industrialization completion. It is noteworthy that this weak decoupling paradoxically reflected a sharp rise in carbon emissions alongside an even stronger surge in value added between 1951 and 1973.

**Fig. 6:**
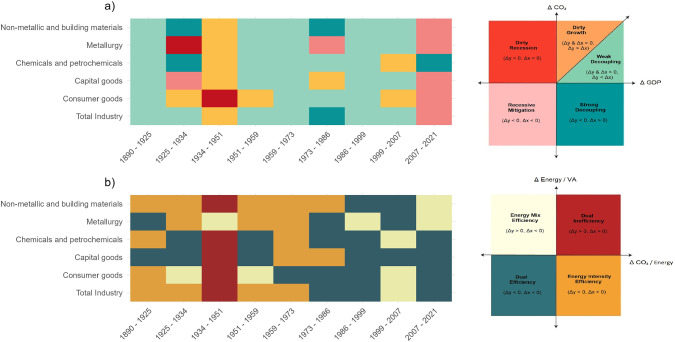
Comparative evolution of value-added growth and CO_2_ emissions (**a**); and energy intensity of value added versus carbon intensity of energy use (**b**). See Supplementary Information SI1 – Table 7 for details on decoupling scenarios. The data for this Figure is available in Zenodo https://zenodo.org/records/17900036

These long-term decoupling dynamics were highly asymmetric across sectors (Fig. 6a). Patterns of weak and strong decoupling were identified before Civil War, attributed to significant gains in energy intensity and partial reductions in carbon intensity, particularly from hydroelectrification in certain activities up to 1934 (Fig. 6b). However, a pattern of "dirty growth" is observed across various activities between 1934 and 1951, within a context of overall dual inefficiency (Fig. 6b). The gradual lifting of economic isolation between 1951 and 1959 was associated with a general reduction in energy costs, resulting in a shift toward weak decoupling.

During the industrial crisis (1973–1986), most activities exhibited a pattern of weak and strong decoupling due to the deindustrialization of heavy industries and the broader restructuring of the industrial base. Weak decoupling became the prevailing trend across industry until 2007. This process also prompted dual efficiency gains (Fig. 6b): first, through the disappearance of many fossil energy–intensive sectors, and later through decarbonization of the energy mix, driven by the gradual integration of carbon–neutral sources and natural gas.

Overall, Spanish industry and its main sectors exhibit a long-term pattern of weak decoupling, which was interrupted during the autarkic period, and followed two distinct trajectories. The first, from 1890 to 1973, was driven primarily by reductions in energy intensity, enabled by the adoption of more efficient energy sources and converters. However, during this same period, the carbon intensity of energy increased due to the growing importance of process-related emissions, generated by highly emissive materials such as artificial cement and steel. From 1986 onward, most industrial sectors shifted toward strategies centered on reducing the carbon intensity of energy consumption—particularly through the growing share of natural gas—while overall energy intensity improvements stagnated (Fig. 5).

### Decomposing the drivers of industrial CO_2_ emissions

**Fig. 7:**
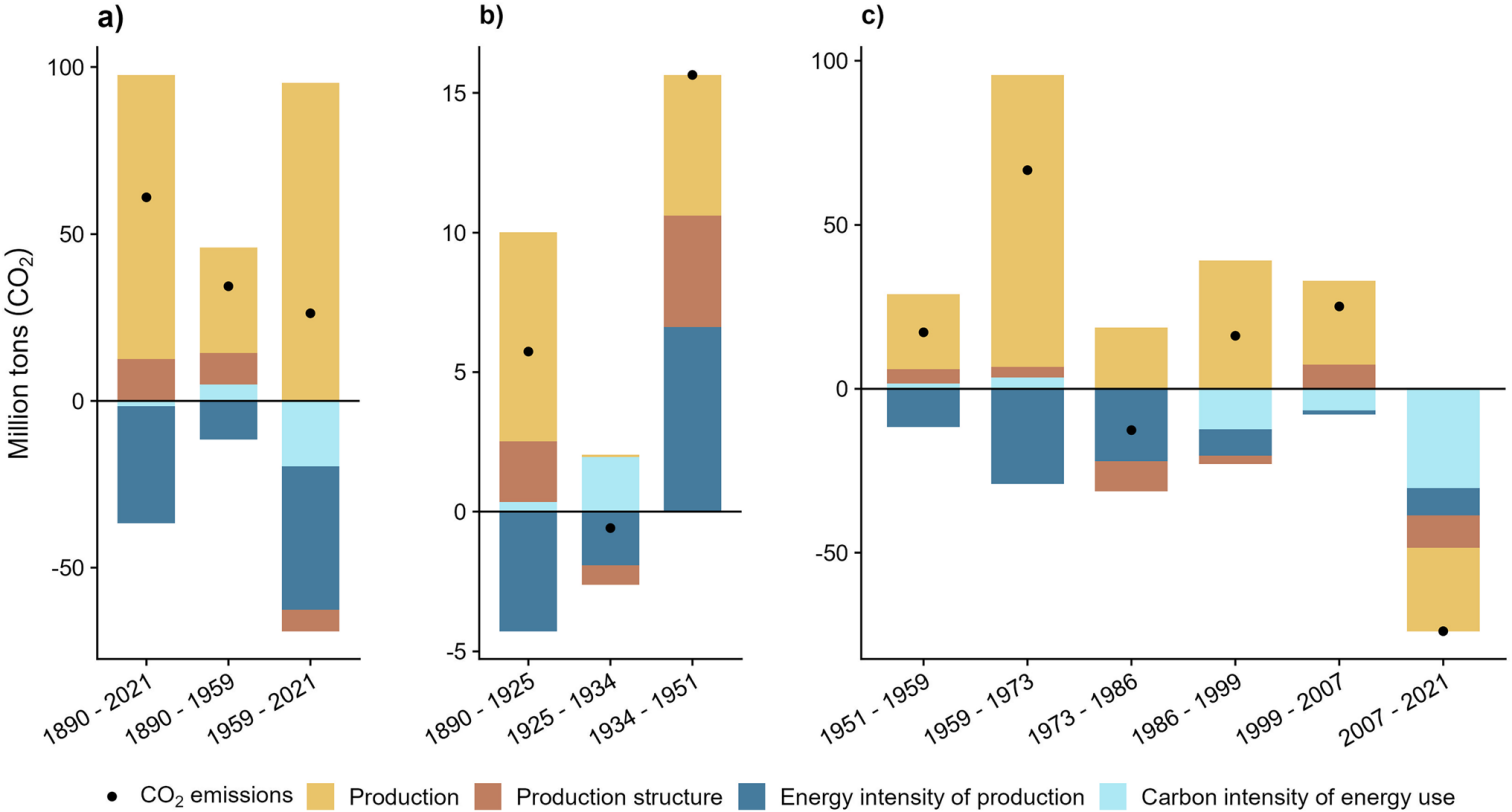
LMDI Decomposition analysis of CO_2_ emissions variation in the Spanish industry over the long-term (**a**); by subperiods from 1890 to 1951 (**b**); and subperiods from 1951 to 2021 (**c**). The data for this Figure is available in Zenodo https://zenodo.org/records/17900036

We now turn to quantifying the relative contribution of the key drivers behind shifting decoupling patterns. Using the LMDI decomposition analysis presented in Fig. 7a, we assess how each factor has influenced the evolution of industrial CO₂ emissions in Spain. Between 1890 and 2021, total emissions increased by 61 Mt. Improvements in energy intensity and in the carbon intensity of energy use resulted in cumulative emission savings of 35 Mt and 1.6 Mt of CO₂, respectively. However, these efficiency gains were insufficient to offset the combined impact of industrial output growth and structural shifts toward more carbon-intensive activities, which contributed respectively to 85 and 12.5 Mt.

Between 1890 and 1959, industrial output emerged as the dominant driver, accounting for 54.5% of total emissions growth. Improvements in energy intensity (20%), structural changes toward more carbon-intensive sectors (16%), and rising carbon intensity of energy use (7.5%) played secondary yet relatively comparable roles. Since 1959, output growth has remained the primary contributor, explaining 57.3% of the increase in emissions, while the role of energy intensity improvements expanded to 28%. Structural change, by contrast, accounted for only 3.5%, reflecting relatively limited shifts toward cleaner activities.

A period-specific analysis further refines these trends. Up to 1925, output growth was the main driver of emissions, while improvements in energy intensity served as the primary mitigating factor (Fig. 7b). During this phase of early industrialization, structural changes increased the carbon intensity of production, accounting for 18% of the emissions rise between 1890 and 1925. Between 1934 and 1951, a sharp increase in energy intensity led to an additional 8 Mt of CO₂—representing 42.8% of the total emissions increase—while the contributions of other drivers remained minor.

From 1951 onward (Fig. 7c), improvements in energy intensity once again enabled substantial mitigation: 12.7 Mt (29.7%) between 1951 and 1959, and 31.1 Mt (24.2%) from 1959 to 1973. Nevertheless, output growth accounted for a larger share of the increase in both periods—56% and 70.7%, respectively.

Between 1973 and 1986, reductions in energy intensity became the dominant factor, explaining 46.2% of the overall decline in CO₂ emissions. Although structural change, within the context of deindustrialization and industrial restructuring policies, accounted for only 11.7%, part of the reduction in energy intensity may be attributed to shifts at the subsectoral level—such as the crisis in shipbuilding and technological advances in electrical equipment within the capital goods sector.

From 1986 to 1999, reductions in the carbon intensity of energy consumption enabled savings of 12.3 Mt (27%) of CO₂. However, industrial output growth remained the main driver, responsible for 63.3% of the emissions increase. During the construction boom (1999–2007), emissions rose primarily due to output expansion (61.5%) and structural shifts toward more carbon-intensive sectors (16.2%). In contrast, gains in energy intensity (8%) and reductions in carbon intensity (5%) played only marginal roles. The impact of low-carbon and carbon–neutral energy sources—such as natural gas and renewables—was partially offset by rising process-related emissions from building materials, which diluted the gains in the energy mix.

After the collapse of the housing bubble in 2008, the sharp decline in emissions was primarily driven by a 41% reduction in the carbon intensity of energy consumption. This shift reflects both the contraction of process-related emissions and the continued decarbonization of electricity generation, increasingly dominated by carbon–neutral sources. The contraction of industrial output accounted for an additional 28.8% of the emissions decline, while reductions in carbon-intensive sectoral activity and improvements in energy efficiency played more limited roles.

### Carbon intensity in Europe’s latecomer industries

**Fig. 8:**
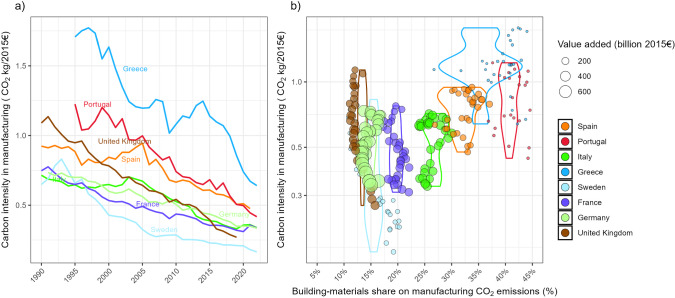
Long-term manufacturing carbon intensity (kg CO₂/2015 €) in selected European countries, 1990–2021 (**a**). Manufacturing carbon intensity (kg CO₂/2015 €) and the building materials share of manufacturing CO₂ emissions (%) in selected countries, 1990–2021 (**b**). Source: see Supplementary Information SI1 – Sect. 5. Note: Building materials share includes direct and indirect emissions from Non-metallic Minerals (NACE, Rev. 2, Division 23) and process-related emissions from cement, lime and glass. The data for this Figure is available in Zenodo https://zenodo.org/records/17900036

The declining but irregular evolution of industrial carbon intensity observed for Spain was also characteristic of other late-joining economies in Southern Europe. As shown in Fig. 8a, the long-term trajectories of Greece and Portugal exhibit higher levels of carbon intensity compared with the more linear downward paths recorded in countries with higher value-added industrial structures, such as Sweden, or in established first-comers including the United Kingdom and Germany.

This gap between first- and late-comers may reflect differences in technological capabilities and innovation uptake, but it is also closely related to structural factors—most notably the relative weight of construction-related activities within national industrial emissions and the slower development of technologically sophisticated manufacturing branches (Fig. 8b).

Southern European countries, where building-materials industries have accounted for between 30–45% of total industrial CO₂ emissions—especially those driven by process emissions—display pronounced volatility and erratic and levels of carbon intensity between the 1990s and the 2010s. In contrast, first-comer economies not only show more stable downward trends but also a markedly lower contribution of construction to industrial emissions, typically below 20%.

## Discussion

### A double delay, a double cost

Industrial growth in Spain since the nineteenth century (Carreras, 2005; Prados de la Escosura, 2017) was shaped by energy scarcity and restrictive institutions, especially during Francoist autarky (Sudrià, 1995). As a result, annual carbon emissions began rising sharply from the 1940s due to the environmental inefficiencies of isolation—an anomaly compared with the global pattern, where major increases appeared only in the 1950s with sustained economic growth (Steffen et al., 2015). Having missed the first wave of industrialization and inheriting high levels of carbon intensity, Spanish industry underwent a compressed expansion after the 1950s in which the rapid adoption of oil-based technologies enabled growth but reinforced structural constraints and produced a surge in CO₂ emissions until 1974. This constituted the first climatic cost of lateness: a carbon-intensive take-off occurring when first-comer European economies were already diversifying technologically.

Structural limitations inherited from Francoism intensified the oil crisis (Sudrià, 2013) and hindered the development of more efficient activities. Spain thus became a latecomer not only to industrialization but also—after 1973—to the adoption of energy-efficient and low-carbon technologies. This second delay compounded the first. Continued reliance on fossil fuels locked the industrial sector into a high-carbon path and slowed diffusion of efficiency measures; industrial oil demand remained subsidized until 1977 (Rubio-Varas & Muñoz-Delgado, 2022), increasing vulnerability to the 1979 shock. Subsequent energy scarcity and the deindustrialization of energy-intensive branches helped reduce energy- and process-related emissions, while the Energy Plans promoted energy diversification (Garrués, 2016, 2022).

The structural bias generated by the late take-off, together with the lack of a high-tech industrial base, steered 1990s investment toward construction-related activities (Maluquer, 2014), pulling industry into a real-estate boom driven by demographic growth, mass-tourism, and speculative capital (Alcántara & Padilla, 2021). Even after industrial crisis (1980–1993), building materials retained a disproportionate weight in the industrial structure—which helps explain why industry experienced the smallest reduction in relative terms by 2005 (Henriques & Kander, 2010)—and, by extension, in emissions. The technological characteristics—high thermal requirements and substantial process emissions—severely constrained reductions in carbon intensity. This configuration generated the second climatic cost of lateness: a renewed acceleration of emissions between 1999 and 2007, driven by a shift toward less efficient activities and strong output growth.

This pattern also helps explain why industrial structures from Southern European latecomers such as Portugal and Greece—and Italy to a lesser extent—remained heavily weighted toward non-metallic and building material activities. Democratic transitions, EU accession, and infrastructure subsidies channeled into roads and public works further reinforced this orientation, which helps explain why these economies continued to exhibit high energy and carbon intensities well into the twenty-first century (Bhattacharyya & Matsumura, 2010; Henriques & Borowiecki, 2017). By contrast, northern first-comers transitioned more steadily toward efficient industrial systems adopting ICT technologies from the 1970s onward (Henriques & Kander, 2010; Kander, 2005; Kander et al., 2013). This earlier and more continuous technological diffusion enabled manufacturing output to expand without generating proportional increases in emissions.

The Great Recession and the collapse of the housing bubble produced a sharp decline in emissions, reinforced by the loss of competitiveness vis-à-vis emerging Asian producers (Baldwin, 2018) and improvements in the carbon intensity of energy consumption (Crippa et al., 2023; Minx et al., 2021). These developments coincided with the consolidation of international and European climate policies whose effects became clearer after the 2015 Paris Agreement.

Spain was thus a latecomer twice over: late to industrialization and late to efficient technological change. Each delay generated a distinct acceleration of emissions, imposing a double ecological burden. The supposed economic advantages of backwardness (Gerschenkron, 1962) became environmental disadvantages once take-off was compressed, fossil-fuel intensive, and technologically dependent. Industrial policy in latecomer economies therefore faces a historical dilemma: reducing environmental impacts while building an efficient and resilient industrial base, a challenge without precedent among late industrializers.

### The hotspots determining carbon intensity

While previous studies have identified the concentration of industrial CO₂ emissions in a few carbon-intensive sectors for the post-1995 period (Cansino et al., 2015; Tarancón & Del Río, 2007), this study shows that such concentration has deep historical roots. As early as 1890–1951, during the phase of weak industrialization, emissions from intermediate goods production already exceeded those from consumer goods. This trend intensified during the rapid industrial expansion of 1959–1973, when three subsectors—cement and lime (23%), iron and steel (21%), and chemicals (14%)—accounted for over half of all industrial emissions.

These sectors share technical characteristics that contribute to their high carbon intensity, and their evolution has been closely tied to historical industrial policy. The autarkic policy of the 1940s and 1950s prioritized heavy industries for military purposes and post–Civil War reconstruction. Industrial policies implemented after 1959 continued to consider these industries as strategically important for the nation (Martín-Aceña & Comín, 1991; De la Torre & García, 2014). The chemical sector contributed to boosting agricultural productivity (Sanchis, 2006), while from 1975 onwards, the petrochemical industry experienced significant expansion (Puig, 2003). Cement production sustained high levels of emissions due to continuous urban and industrial growth—first under state-led development plans (Cardesín & Mirás, 2015; Díaz & Parreño, 2006), and later during the real estate boom (Alcántara & Padilla, 2021; Naredo, 2009).

Historically, different models have produced contrasting environmental outcomes: the Francoist emphasis on output prioritized fossil-fuel-intensive growth despite Spain’s resource constraints, while the market liberalization of the 1980s and 1990s continued to favor high-emission sectors (Catalán, 2012; Maluquer, 2014). Any industrial policy must consider, on the one hand, its impact on energy demand: whether it will concentrate activity in energy-intensive sectors and whether the energy used will be carbon–neutral. However, energy demand is only part of the picture. On the other hand, as process emissions—unrelated to energy use—account for roughly one fifth of total industrial emissions—the design of an industrial model, therefore, must address process-based emissions, crucially in late-joiners highly populated and with a dynamic construction sector. Effective mitigation strategies must target these emission-intensive "hotspots," accounting for their technical and structural complexity (Bataille et al., 2018; Wen et al., 2022).

### Soaring production and overshadowed efficiency

Apart from the autarkic period, Spain’s industrial energy intensity has declined substantially over the long term. Since 1984, the carbon intensity of energy use has also decreased significantly. However, the growth of industrial production has consistently outpaced these efficiency gains, making output expansion the main driver of rising CO₂ emissions. This trend reveals two key historical and policy implications.

First, improvements in energy efficiency have enabled only a relative decoupling between output and emissions—insufficient to reverse the upward trajectory of emissions during periods of industrial expansion. This pattern is consistent with rebound effects, whereby efficiency gains lower the effective cost of energy services, stimulating additional production and consumption that partially or fully offset the expected energy savings (Greening et al., 2000; Hertwich, 2005). This mechanism operates at multiple scales—from the direct consumption response within a given activity, through indirect income and substitution effects that redirect efficiency savings toward other energy-using activities, to broader economy-wide dynamics in which efficiency-driven productivity growth fuels aggregate output and energy demand (Sorrell, 2009; York et al., 2022). Although our LMDI results cannot disentangle these channels, reveal a persistent pattern across branches and subperiods in which declining energy intensity coexisted with expanding output—consistent with rebound mechanisms operating at more than one of these scales. Between 1951 and 1973, declining energy intensity coincided with demographic growth, rising incomes, and stable energy prices (Carreras & Tafunell, 2005), conditions that likely amplified the rebound (Font & van der Voet, 2014). As a result, the most rapid emissions growth occurred during phases of weak decoupling rather than under overtly carbon-intensive growth models (Santarius, 2012). Strong decoupling has remained limited to isolated, sector-specific cases.

Second, each historical phase followed distinct cost-saving strategies. Between 1890 and 1980, reductions in carbon intensity stemmed mainly from technological and structural changes improvements. Since 1986, decarbonization of the energy mix has been the dominant mitigation driver, while energy efficiency remained stagnant. This shift is relevant for emission-intensive sectors. As maximum efficiency has not yet been achieved, there remains significant untapped potential. In steel, for example, emissions could be significantly reduced through greater use of scrap steel or biofuels (Suopajärvi et al., 2018; Zhang et al., 2025). Closing material life cycles is critical—not only for reducing domestic emissions, but also those embedded in imports (Krausmann et al., 2017). Circular economy strategies—such as reducing per capita floor area, extending building lifespans, or substituting cement and steel with lower-carbon materials like wood—offer promising mitigation potential (Pauliuk et al., 2024).

Importantly, some of the largest reductions in carbon intensity occurred during phases of deindustrialization, especially in high-emission sectors. While this structural shift may reflect domestic changes, it could also result from outsourcing polluting industries abroad (Leisner et al., 2023), turning Spain into a net importer of emissions—22% of which are estimated to be embedded in foreign production (Friedlingstein et al., 2023). This issue remains a pressing question for climate policy research.

## Limitations and future research

This study presents several limitations, among which the following stand out.

First, it provides production-based information on industrial emissions. Since 1990, consumption-based estimates have been available through MRIO approaches. However, it is not currently possible to replicate such an approach for the long-term period analyzed in this paper. Future research should address the impact of emission transfers and actual carbon footprints, not only because of their current relevance —given deindustrialization and imports from countries such as China— but also due to their likely significance in the past, when economies were highly dependent on the then “workshops of the world,” such as the UK (Kander et al., 2017).

Second, the study focuses on production-based industrial CO₂ emissions through decomposition analysis. It does not account for embodied emissions, which could be examined with input–output methods. This is particularly relevant for mass tourism—especially real estate and hospitality demand—which likely contributed to emissions in construction and energy-intensive sectors. Allocating such emissions over the long term remains a challenge. More broadly, a consumption-based approach could also help assess whether the environmental disadvantages documented here for latecomers partly reflect their comparatively limited capacity to outsource pollution-intensive production abroad—a structural asymmetry between early industrializers and latecomers that warrants further comparative analysis.

Third, the study suggests that latecomers enter the late twentieth century with an industrial structure and capital stock shaped by earlier, fossil-fuel-intensive development paths, and thus benefit less—and later—from the diffusion of more efficient technologies that had been progressively adopted in core European industries since the 1970s. How backwardness in other late-industrializers affected carbon intensity in earlier stages require further research.

Finally, this study relies on estimates subject to a considerable degree of uncertainty due to methodological limitations. To enhance the accuracy and reliability of long-term sectoral environmental accounting, greater efforts should be directed toward reducing this range of uncertainty.

## Conclusions

This article provides a reconstruction of CO₂ emissions by process, source, and subsector for the period 1890–2021 in Spain—the first to offer this level of disaggregation extending back to the nineteenth century for any country. Industrial CO₂ emissions in Spain increased 12-fold between 1890 and 2021, with the sharpest surge occurring between 1950 and 1974, reflecting the country’s late but accelerated industrialization. Cheap oil, trade liberalization, and industrial electrification triggered a rapid expansion of fossil-fuel-sourced emissions just as other Western economies began to seek energy- and carbon-saving strategies. This double delay—both in industrial takeoff and in the adoption of saving strategies—locked Spain into a carbon-intensive path. Since 1980, emissions declined due to the contraction of heavy industries and shifts toward less emissive energy sources, and emerging climate policies. However, the post-industrial structure that followed remained fragile and increasingly tied to construction dynamics, reinforcing environmental vulnerabilities.

Throughout this trajectory, emissions have been consistently concentrated in a few carbon-intensive sectors—cement, steel, and chemicals—and efficiency gains have not kept pace with output growth, limiting decoupling and even pointing to persistent rebound effects. The most significant reductions occurred during economic crisis and external shocks rather than through proactive mitigation. These findings underscore the need for targeted, sector-specific decarbonization strategies, including material circularity, electrification with carbon–neutral sources, and structural shifts in demand. For late-industrializing economies like Spain, the challenge lies in breaking from the historical legacy of carbon lock-in and technological dependency while avoiding the outsourcing of environmental impacts.

## Supplementary Information

Below is the link to the electronic supplementary material.

Supplementary file1 (DOC 13555 KB) This supporting information document is organized into five sections. The first delimits and justifies the unit of analysis and discusses the reliability of the historical sources employed. The second describes the harmonization procedures applied to industrial sectors and energy consumption in the construction of the time series. The third outlines the methods used to estimate final energy consumption, the conversion factors applied to derive primary consumption and energy- and process-sourced CO₂ emissions, and the sensitivity analysis conducted to test the robustness of these estimates. The fourth explains the variables used in the decomposition analysis and in the decoupling analysis, and discusses the chosen periodization, contrasting it with alternatives based on structural breaks analysis, simple five-year averages, and five-year moving averages. Finally, the fifth describes the sources and methods employed to estimate industrial carbon intensity and sectoral emissions share in other European countries from 1990.

## Data Availability

All data and R scripts required to replicate the figures presented in this article are available in [Zenodo](https:/zenodo.org/records/17900036).

## Footnotes

1 The literature has identified the presence of autocorrelation in long-run series of energy use, emissions, and economic activity, both within and across sectors (Moutinho et al., 2017). Adjacent observations are not independent but reflect persistent dynamics. These should be interpreted as cumulative trajectories shaped by both external shocks and the underlying inertia of industrial activity, rather than as statistically independent breaks.
