# Supplementary material for: The climate cost of lateness: decomposing historical drivers of CO2 industrial emissions during Spanish industrialization and deindustrialization, 1890–2021
Source: J Ind Ecol. 2026 Apr 20;30(3):1129–45. doi: 10.1007/s44498-026-00077-1 (PMC13342420; doi:10.1007/s44498-026-00077-1)
Supplement: Supplementary file 1 — Supplementary file1 (DOC 13555 KB) This supporting information document is organized into five sections. The first delimits and justifies the unit of analysis and discusses the reliability of the historical sources employed. The second describes the harmonization procedures applied to industrial sectors and energy consumption in the construction of the time series. The third outlines the methods used to estimate final energy consumption, the conversion factors applied to derive primary consumption and energy- and process-sourced CO₂ emissions, and the sensitivity analysis conducted to test the robustness of these estimates. The fourth explains the variables used in the decomposition analysis and in the decoupling analysis, and discusses the chosen periodization, contrasting it with alternatives based on structural breaks analysis, simple five-year averages, and five-year moving averages. Finally, the fifth describes the sources and methods employed to estimate industrial carbon intensity and sectoral emissions share in other European countries from 1990. [file 44498_2026_77_MOESM1_ESM.doc]

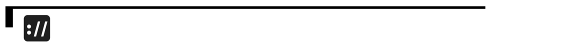


SUPPORTING INFORMATION FOR:

Sanjuán-Ruiz, Á., Infante-Amate, J. & Aguilera, E. The climate cost of lateness. Decomposing historical drivers of CO_2_ industrial emissions during Spanish industrialization and deindustrialization, 1890-2021. *Journal of Industrial Ecology.*

***
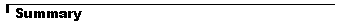
***

This supporting information document is organized into five sections. The first delimits and justifies the unit of analysis and discusses the reliability of the historical sources employed. The second describes the harmonization procedures applied to industrial sectors and energy consumption in the construction of the time series. The third outlines the methods used to estimate final energy consumption, the conversion factors applied to derive primary consumption and energy- and process-sourced CO₂ emissions, and the sensitivity analysis conducted to test the robustness of these estimates. The fourth explains the variables used in the decomposition analysis and in the decoupling analysis, and discusses the chosen periodization, contrasting it with alternatives based on structural breaks analysis, simple five-year averages, and five-year moving averages. Finally, the fifth describes the sources and methods employed to estimate industrial carbon intensity and sectoral emissions share in other European countries from 1990.


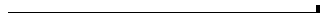


**Authors:**

Ángel Sanjuán-Ruiz,^1^ Juan Infante-Amate,^2^ Eduardo Aguilera,^3^

**Institutions:**

^1^ Department of Geography, History and Philosophy, Pablo de Olavide University, Seville, Spain.

^2^ Department of Economic Theory and History, University of Granada, Granada, Spain.

^3^ Institute of Economics, Geography and Demography, Spanish National Research Council (CSIC), Spain.

**Corresponding Author:** Ángel Sanjuán-Ruiz. [asanrui@upo.es](mailto:asanrui@upo.es)

**ORCID:** Ángel Sanjuán-Ruiz: 0000-0003-1031-1219; Juan Infante-Amate: 0000-0003-1446-7181; Eduardo Aguilera: 0000-0003-4382-124X.

1. **BOUNDARIES AND SOURCES**
   1. **What is included in this research**

Although other greenhouse gases have had historically significant impacts (Infante-Amate et al., 2024), this study focuses exclusively on CO₂ emissions, which in 2022 accounted for about 90% of Spain’s total GHG emissions (direct and indirect) and up to 97% within the industrial sector (Inventario Nacional de Emisiones, 2024).

CO₂ emissions are generally classified into five broad categories: energy systems, industry, buildings, transport, and AFOLU (Agriculture, Forestry and Other Land Use) (IPCC, 2022). However, several studies reallocate indirect emissions—particularly those from electricity generation—to the sectors where energy is ultimately consumed (Lamb et al., 2021; Liu et al., 2015; Rissman et al., 2020; Shan et al., 2019; Shao et al., 2016). For industry, while some databases provide subsectoral desaggregation, they typically exclude electricity-related indirect emissions (Crippa et al., 2023; Minx et al., 2021). Consequently, very few datasets offer consistent coverage of both direct and indirect CO₂ emissions at the subsectoral level. This paper builds on an original database that disaggregates industrial CO₂ emissions in Spain by source—direct, indirect, and process-related—and by sector.

Regarding sectoral coverage, the analysis is restricted to manufacturing industries (NACE Rev. 2, Section C). The extractive industries and construction (NACE Rev. 2, Sections B and F) are excluded due to data limitations. The energy sector and utilities (NACE Rev. 2, Sections D and E) are also excluded, given the substantial methodological challenges of reconstructing long-term series that differentiate between the sector’s own energy use and inputs consumed in energy transformation. Over the long run, emissions estimates are reported for seven groups of activities: iron and steel, non-ferrous metals, cement and concrete, bricks and glass, chemicals, capital equipment, and consumer goods (see harmonization details in section 2.1).

- 1. **Historical sources and reliability**

Before 1958, industrial statistics in Spain were scarce. An exception is the *Estadística Minera y Metalúrgica* (EMME), published since 1861, which reports physical production data and other socioeconomic variables, complemented by qualitative notes written by mining and industrial engineers. Consequently, reconstructing annual series prior to 1958 requires combining multiple primary and secondary sources and relying on explicit assumptions to estimate energy consumption.

The *Estadística sobre el consumo de carbones por las diferentes industrias* (ECC) provides sectoral coal consumption data beginning in 1932. For earlier years, fragmented evidence is available from specialized outlets such as the *Revista Minera*, which occasionally published sector-specific coal consumption data (Oriol, 1894; Lucio, 1924). The EMME also contains qualitative reports by mining engineers on coal and charcoal consumption per unit of output at the firm level. In addition, the *Anuario Estadístico de España* (AEE) records industrial electricity consumption between 1900 and 1956, and coal consumption for 1948 and 1951.

From 1958 onwards, annual statistics on industrial production and energy use by sector were published, albeit with varying levels of sectoral disaggregation and accounting methodologies. The *Estadística Industrial de España* (EIE), launched in 1958, provides final consumption data for 17 energy carriers across more than 100 activities. From 1978, the *Encuesta Industrial* (EI) reports final consumption for seven energy carriers across 86 activities. Since 1990, official balances from the *Instituto para la Diversificación y Ahorro Energético* (IDAE), aligned with Eurostat’s energy balance methodology, have provided data for twelve broad groups. Additionally, the *Encuesta de Consumos Energéticos* (ECE) reports monetary expenditure on energy carriers for the period 2001–2021.

The reliability and robustness of these sources have been the subject of debate. Coll and Sudrià (1987) corrected the sectoral coal consumption figures reported in the ECC and *Revista Minera*, highlighting inconsistencies with national apparent coal consumption. Bartolomé (2007) re-examined AEE data and cross-checked it against alternative publications on electricity use in electro-intensive industries (Errandonea, 1935).

To mitigate these limitations, several adjustments were introduced. First, we adopted the sectoral corrections proposed by Coll and Sudrià (1987) for coal, by Iriarte and Infante-Amate (2019) for firewood, and by Bartolomé (2007) for electricity. For the years after 1936, electricity consumption data from the AEE (INE, 1951) were used directly.

By contrast, energy consumption figures reported in the EIE and EI have been criticized for their limited accuracy (Zapata, 1996; Parejo, 2001; Carreras, 2005). In the case of the EIE, problems are particularly pronounced in metallurgical sectors and local-scale industries such as carpentry workshops and wineries (Llopis & Fernández, 1997), which complicates long-term reconstructions. Coverage improved substantially with the EI, making it the main source for sectoral analysis during 1978–1990, despite acknowledged limitations (García et al., 1994). Among these, the survey systematically covered only establishments with more than 20 employees, while the rest were estimated through sampling. For these reasons, successive processes of correction and harmonization were necessary.

1. **Long-term harmonization procedures**
   1. **Sectoral aggregation**

A key methodological challenge in reconstructing long-term time series is sectoral harmonization. Energy data are reported at different levels of aggregation and in varying measurement units. To address this heterogeneity, all sectoral classifications in the sources were systematically mapped, and their historical evolution was traced. Whenever activities were merged in the original classifications, they were aggregated consistently across the entire series.

| **Level 4** | **Level 3** | **Level 2** | **Level 1** | **NACE Rev. 2 code** |
| --- | --- | --- | --- | --- |
| Capital Equipment | Capital goods | Metal goods and machinery | Metal goods and machinery | Divisions 25, 26, 27 and 28 |
|  |  | Transport equipment | Aircrafts | Groups 30.3 and 30.4 |
|  |  |  | Railway Materials | Groups 30.2 |
|  |  |  | Shipbuilding | Group 30.1 |
|  |  |  | Transport equip. | Divisions 29 and 30* |
|  |  |  | Vehicles | Group 29 |
| Chemicals and petrochemicals | Chemicals and petrochemicals | Chemicals and petrochemicals | Chemicals | Divisions 19, 20 and 21, except Group 20.6 |
| Consumer goods | Consumer goods | Food | Animal Feed | Group 10.9 |
|  |  |  | Beverage | Division 11 |
|  |  |  | Dairy Industry | Group 10.5 |
|  |  |  | Fish Preservation | Group 10.2 |
|  |  |  | Food | Division 10 and 11* |
|  |  |  | Meat Industry | Group 10.1 |
|  |  |  | Milling | Group 10.6, 10.7, 10.8 |
|  |  |  | Oils & Fats | Group 10.4 |
|  |  |  | Tobacco | Division 12 |
|  |  |  | Vegetable Preserves | Group 10.3 |
|  |  | Other consumer goods | Furniture | Division 31 |
|  |  |  | Plastics & Rubber | Division 22 |
|  |  |  | Other consumer goods | Division 32 |
|  |  | Pulp, paper and cardboard | Other papers | Division 17 and 18* |
|  |  |  | Paper Goods | Group 17.2 |
|  |  |  | Publishing & Arts | Division 18 |
|  |  |  | Pulp & Paper | Group 17.1 |
|  |  | Textil and leather | Artificial Fibers | Group 20.6 |
|  |  |  | Footwear | Group 15.2 |
|  |  |  | Leather Goods | Group 15.1, Class 14.11 |
|  |  |  | Other textiles | Division 13, 14 and 15* |
|  |  |  | Textiles & Spinning | Division 13 and 14, except class 14.11 |
|  |  | Wood | Other woods | Division 16* |
|  |  |  | Wood Industry | Group 16.2 |
|  |  |  | Wood Sawing | Group 16.1 |
| Metallurgy | Iron and steel | Iron and steel | Iron and steel | Groups 24.1, 24.2 and 24.3, Divisions 24.51 and 24.52 |
|  | Non-ferrous metals | Non-ferrous metals | Non-ferrous metals | Group 24.4, Divisions 24.53 and 24.54 |
| Non-metallic and building materials | Bricks, Stones & Glass | Bricks, Stones & Glass | Other building materials | Division 23* |
|  |  |  | Ceramic Products | Groups 23.2, 23.3, 23.4 and 23.9 |
|  |  |  | Glass | Group 23.1 |
|  |  |  | Natural Stones | Group 23.7 |
|  | Concrete & Lime | Concrete & Lime | Concrete & Lime | Group 23.5 and 23.6 |

*Table 1. Harmonization and aggregation levels of industrial sectors along historical sources. (*) These subsectors include non-defined products before 1958 that do not correspond to specific NACE Rev.2 groups, but just with broad divisions.*

To implement this harmonization, we defined several levels of aggregation corresponding to the maximum degree of disaggregation allowed by data availability (Table 1). The first level reflects the most detailed disaggregation attainable, covering the period 1960–2021. The second level follows Eurostat’s sectoral classification. The third level represents the maximum disaggregation possible when extending the energy and emissions series back to 1890. Finally, the fourth level aligns our classification with that of Gross Value Added (GVA) from Prados de la Escosura (2017), which is the basis for the decomposition analysis used in the paper.

- 1. **Energy use harmonization**

Final energy consumption has been categorized into six energy carriers: coal, oil, natural gas, electricity, hydropower, and biomass. The sources report this data in different measurement units and with varying levels of detail depending on the energy carrier (Table 2). To ensure comparability, all values were converted into gigajoules (GJ) using standardized energy density factors for each carrier (see section 3.3).

| **Energy carrier** | **ECC and others 1890-1934** | **AIE 1948 and 1951** | **EIE 1958-1977** | **EI 1978-1992** | **IDAE 1990-2021** | **ECE 2001-2021** |
| --- | --- | --- | --- | --- | --- | --- |
| **Coal** | Coal | Hard coal | Hard coal | Coal | Hard coal | Coal and derivates |
|  |  | Anthracite | Anthracite |  | Anthracite |  |
|  |  | Lignite | Lignite |  | Lignite |  |
|  |  | Cok | Coal |  | Coal |  |
|  |  | Agglomerates | Coke gas |  | Agglomerates |  |
|  |  |  | Manufactured gas |  | Cok |  |
|  |  |  |  |  | Coke gas |  |
|  |  |  |  |  | Manufactured gas |  |
| **Oil** | - | - | Fuel oil | Fuel oil | Fuel oil | Fueloil |
|  |  |  | LPG | LPG | LPG | Gasoil |
|  |  |  | Gasoil | Gasoil A and C | Gasoil | Others |
|  |  |  | Gasoline | Gasoline | Gasoline |  |
|  |  |  |  |  | Querosene |  |
|  |  |  |  |  | Petroleum coke |  |
|  |  |  |  |  | Others |  |
| **Natural gas** | - | - | Natural gas | Natural and manufactured gas | Natural gas | Natural gas |
| **Electricity** | Electricity | Electricity | Electricity | Electricity | Electricity | Electricity |
| **Biomass** | Firewood | Firewood | Firewood | - | Biomass | Biofuels |
|  |  |  |  |  | Biogas |  |
|  |  |  |  |  | Biofuels |  |

Table 2. Harmonization and aggregation levels of energy carriers along historical sources.

1. **Estimation procedures**
   1. **Final consumption estimation**

The large number of gaps in the statistical records prior to 1958 required combining different estimation methods to reconstruct the long-term series, each with varying degrees of robustness. The most critical and demanding estimations concern final energy consumption, as these form the basis for calculating CO₂ emissions.

- - 1. **Biomass and hydropower consumption**

The estimation of so-called traditional energy sources has long posed a major challenge in the literature, given the absence of official statistical records during periods when they accounted for the bulk of energy consumption (Fouquet, 2008; Kander et al., 2013).

In this study, we estimated charcoal and firewood consumption in iron and steel, non-ferrous metals, cement and concrete, bricks, stone, and glass using observations of sector-specific energy consumption (SEC) drawn from a variety of primary and secondary sources (Table 3). These observations were smoothed using a local regression (LOESS), which captures the annual trend in SEC. Final biomass consumption by sector was then derived by applying the estimated SEC trend to the physical production series reported by Nadal (2003). For iron and steel, provincial-level data allow us to reconstruct annual consumption at both national and regional scales, reflecting the well-documented differences in efficiency associated with coal quality and blast furnace size.

| **Product** | **Firewood or charcoal** | **Cok** | **Other coals** | **Electricity** |
| --- | --- | --- | --- | --- |
| **Pig Iron^1-8^** | 55 | 153 | 25 |  |
| **Wrought Iron^1,3,10-11^** | 38 |  | 18 |  |
| **Steel^1,5,6,12-15^** |  | 35 | 149 | 14 |
| **Rolled Iron and Steel^1,14,15^** |  |  | 486 |  |
| **Cements^1^** | 6 | 2 | 34 |  |
| **Bricks and glass^1^** | 4 | 4 | 27 |  |
| **Lead^1^** | 2 | 9 | 10 | 2 |
| **Zinc^1^** | 1 |  | 29 | 2 |
| **Cupper^1^** | 15 | 25 | 21 | 1 |
| **Aluminium^1^** |  |  |  | 1 |
| **Mercury^1^** | 71 | 39 | 67 |  |
| **Calcium Carbide^1^** |  | 2 | 2 | 2 |
| **Ammonium Sulphate^1^** |  |  | 3 | 6 |
| **Ammonia^1^** |  |  |  | 1 |
| **Sulfuric Acid^1^** |  |  |  | 2 |
| **Nitric Acid, Superphosphates and others^1^** |  |  |  | 4 |
| **Caustic Soda^1^** |  |  | 1 |  |

*Table 3. Observations of Specific Energy Consumption (SEC). Source: (1) EMME (various years); (2) Anés, 1988; (3) Fernández, 1985; (4) Bilbao y Fernández, 1988; (5) Escudero & Parejo, 2015; (6) Escudero, 2005; (7) Fernández de Pinedo, 1988; (8) Ojeda, 1985; (9) Bilbao, 1985; (10) Corbera, 2001; (11) Nadal, 1984; (12) Anés & Ojeda, 1983; (13) Bilbao, 1984; (14) González Portilla, 1985; (15) Houpt, 1998.*

Infante et al. (2022) provide estimates of industrial biomass consumption for 1860 and 1900 at the aggregate level. The residual difference was allocated to the Consumer Goods sector, consistent with their qualitative evidence indicating that most biomass use was concentrated in agri-food and wood-related industries. Since this only allows a subsectoral estimate for 1900, consumption for the selected benchmarks was derived by extrapolating from the national trend in annual biomass use.

For the period 1958–1976, biomass consumption data are taken from the EIE. As the EI does not report biomass, sectoral consumption between 1976 and 1989 was linearly interpolated. This procedure does not materially affect the results, given that biomass accounted for only 2% of total energy consumption in 1976 and 5% in 1990.

Hydropower use in 1890 was estimated from installed capacity reported in the EMME for metallurgy, chemicals, and construction materials, and from Nadal (2003) for the remaining sectors. Following previous studies (Kander, 2002; Malanima, 2006), we assume a 30% efficiency rate and 300 operating days per year. The resulting estimate represents less than 0.1% of total industrial energy use, likely due to water scarcity, the predominance of labor-intensive industries, and the relatively low horsepower per installed unit in agri-food activities, which predominated in Spanish industrial structure.

- - 1. **Coal consumption**

Between 1890 and 1934, data on final coal consumption are available at varying levels of sectoral aggregation (Table 4, from Coll & Sudrià, 1987). To refine this disaggregation, we applied the same procedure used for biomass to estimate coal consumption in heavy industries. The residual difference between total industrial coal use and the estimated amounts for these sectors was allocated to Capital Equipment and Consumer Goods.

| SECTOR | **Ca. 1890** | **1894** | **Ca. 1925** | **1932** | **1933** | **1934** |
| --- | --- | --- | --- | --- | --- | --- |
| Metallic goods | 1100 | - | 1300 | 67.2 | 66 | 62.3 |
| Iron and Steel |  | 592 |  | 926.9 | 724.3 | 867.4 |
| Non-ferrous metals |  | 357 | 200 |  | 123 |  |
| Mining |  | - | 250 | 221.3 | 248.9 | 253.1 |
| Sugar | 915 | - | 600 | 350.1 | 291.1 | 345 |
| Cement |  | - |  | 482.7 | 403.4 | 374.4 |
| Glass |  | - | 200 | 113.7 | 103 | 84 |
| Ceramics |  | - |  | 77.4 | 69.7 | 74.2 |
| Paper |  | - | 700 | 135 | 132.7 | 126.9 |
| Others |  | - |  | 119.4 | 112.8 | 117.8 |
| Chemicals |  | - |  | 153.1 | 153.2 | 174.1 |
| Textil |  | - |  | 223.6 | 225 | 223.8 |

*Table 4. Corrected sectoral distribution of coal consumption in Spain (thousand tons). Source: Coll & Sudrià, 1987: 364.*

For 1948 and 1951, data are drawn from the AEE (1951), which reports consumption of bituminous coal, anthracite, lignite, coke, and coal briquettes for 29 sectors. Between 1958 and 1989, the EIE and EI provide annual coal consumption by sector, but exclude coke. Coke use in the steel industry was therefore estimated from the production of metallurgical coke, reported in the same source.

- - 1. **Electricity consumption**

Regarding electricity consumption, Bartolomé (2007) provides annual data for the entire industry between 1901 and 1936, as well as an estimate for electro-intensive industries in 1935. Using the same procedure described for coal, SEC was estimated for heavy industries, with the residual difference allocated to the remaining sectors. The limitation in this case is that the residual cannot be clearly attributed to either Capital Equipment or Consumer Goods. However, this does not affect the results for two reasons: first, indirect industrial emissions before 1934 accounted for only about 2% of the total (see Fig. 2a in the main paper); and second, the decomposition analysis relies on aggregate energy intensity, while structural change is measured using sectorally disaggregated Gross Value Added (GVA).

For 1948 and 1951, the AEE (1951) reports electricity consumption in two categories: electro-intensive industries and all others. To refine this classification, we estimated the shares of electro-chemistry and electro-metallurgy within the electro-intensive group and calculated their proportion of the AEE total. Consumption in the remaining industries was disaggregated by applying the sectoral distribution reported in the 1956 yearbook, which distinguishes 22 sectors. Although this assumes that the sectoral distribution of electricity consumption remained unchanged over a five-year span, the stability of electricity’s share in the final energy mix (7–8%) and the relatively small contribution of indirect emissions (about 5% of the total) support the validity of this assumption as this barely affects results.

Between 1958 and 1989, data are available from the *Estadística Industrial de la Energía Eléctrica* (EIEE), which reports 16 sectors, while the EIE and EI report 31. Sectoral trends were compared across these sources, revealing inconsistencies in some cases. Because EIEE data show better continuity with Eurostat’s official balances from 1990 onward, sectoral consumption figures from EIEE were adopted. To preserve the greater level of sectoral detail provided by the EIE and EI, we calculated the relative shares of each industrial division within broader sectors and applied these proportions to the EIEE data.

- - 1. **Oil consumption**

Regarding oil consumption, no sectoral data is available prior to 1958, when oil use represented 7% of industrial consumption and 10% of national oil consumption (Gales et al., 2007). To reconstruct earlier values, we extrapolated backward following the trajectory of national oil consumption, which implicitly assumes that the industrial share of total oil use remained constant since records began. Given that oil consumption was negligible before 1950, this assumption does not materially affect the results.

- - 1. **Subsectorial estimation from 1990**

Since no data on final energy consumption by subsector are available after 1990, we adopted the following procedure. From that year onward, the official energy balances of IDAE (2024), based on Eurostat’s methodology (2019), report final energy consumption for only twelve industrial sectors. By contrast, national accounts provide data on monetary expenditure by energy type for 97 subsectors between 1958–1992 and 2001–2021. We reconstructed subsectoral trends in energy expenditure, linearly interpolating the gap between 1993 and 2000. These subsectors were grouped following NACE Rev. 2 criteria, and the share of energy expenditure within each sector was calculated. These shares were then applied to the IDAE data to estimate final energy consumption by carrier.

This method has two main limitations. First, the method applies relative expenditure patterns within each sector under the assumption that price changes affect subsectors (e.g., cement and ceramic manufacturing under non-metallic minerals) equally. Second, it assumes that the subsectoral distribution of energy use changed linearly across the interpolated years (1993 and 2001).

- 1. **Process-sourced emissions**

The *Inventario Nacional de Emisiones* (2024) provides annual process-related emissions data by product from 1990 onward. These data were combined with physical production statistics from 1890 to calculate emission factors in kg of CO₂ per unit of output. The 1990 benchmark was then extrapolated backward, with the remaining series estimated by applying these factors to the historical production trends of steel, cement, sodium carbonate, nitric acid, and ammonia, which account for most of process-related carbon emissions.

- 1. **Conversion factors**

Using average conversion factors, we calculated primary energy consumption (Table 5). For electricity, adjustments account for changes in the generation mix, improvements in efficiency, and distribution losses (Fig. 9). For details on the methods used to estimate primary electricity, see Aguilera et al. (2019).

| Energy carrier | Unit | Final MJ / unit consumed | Primary MJ / final MJ | kgCO_2_ emissions / Primary MJ |
| --- | --- | --- | --- | --- |
| Electricity | kwh | 3.6 | Depending on annual electric mix (Aguilera et al., 2019) | |
| Firewood | kg | 15.3 | 1.05 | 0 |
| Charcoal | kg | 29.3 | 3.3 | 0 |
| Coke | kg | 28 | 1.5 | 98.33 |
| Other coals | kg | 22.4 | 1.084 |
| LPG | m3 | 28 | 1.182 | 77.25 |
| Gasoline | liter | 34 |
| Gasoil | liter | 35.8 |
| Fueloil | kg | 42 |
| Natural gas | m3 | 37 | 1.195 | 56.04 |

*Table 5. Main conversion factors, adapted from Eurostat (2019) and IPCC (2022).*

*
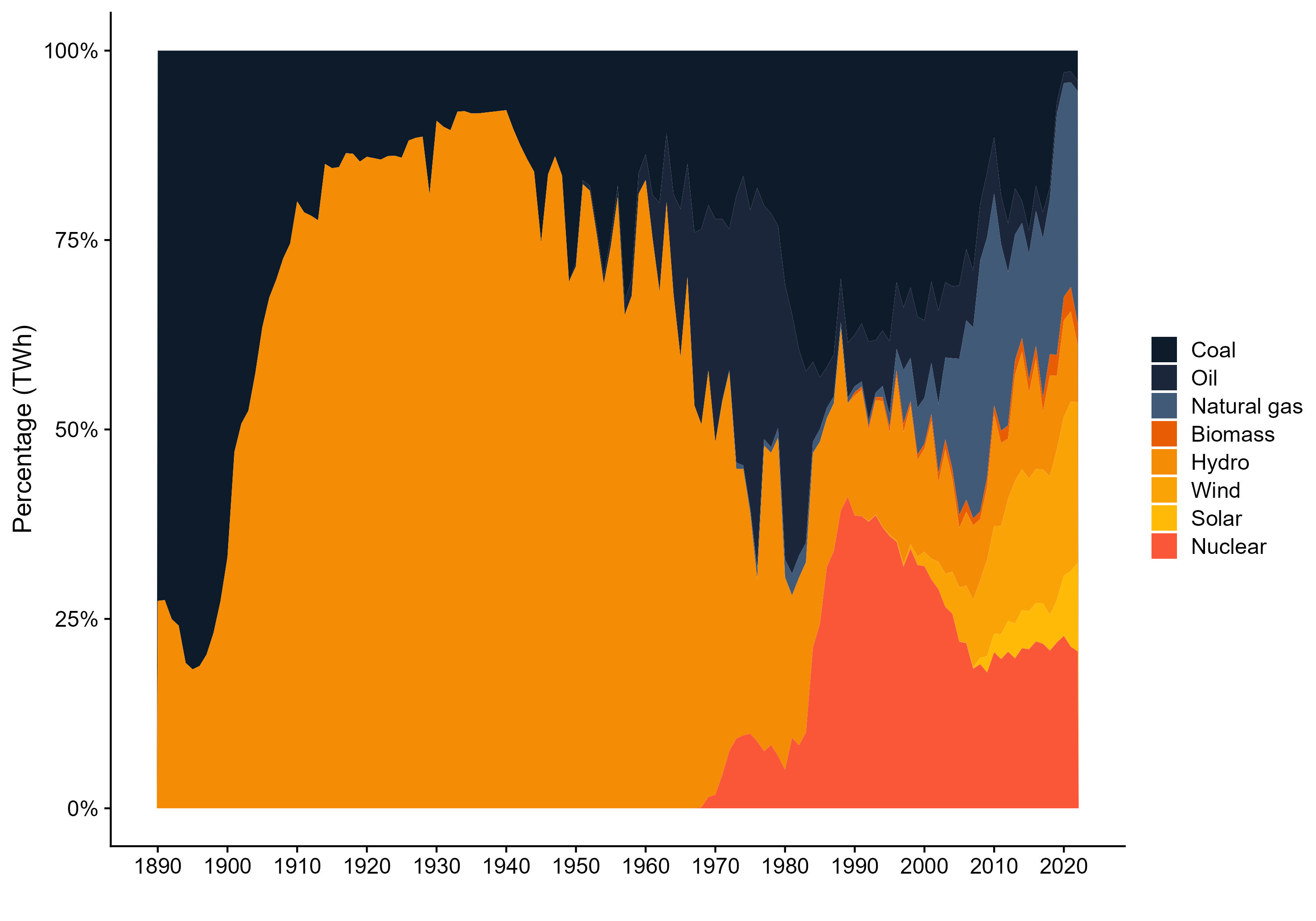
*

*Fig. 9 Energy mix for electricity generation in Spain. Notes: Data extracted from the estimates of Aguilera et al. (2019). Includes losses from pumping and electricity distribution*

Primary energy series were converted into CO₂ emissions using standardized conversion factors from the IPCC (2022). In line with the *Inventario Nacional de Emisiones* (2024), biomass consumption is treated as CO₂-neutral due to its biogenic origin.

As a result, the dataset provides subsectoral series of process- and energy-related emissions for the period 1960–2021 (Figs. 10 and 11).


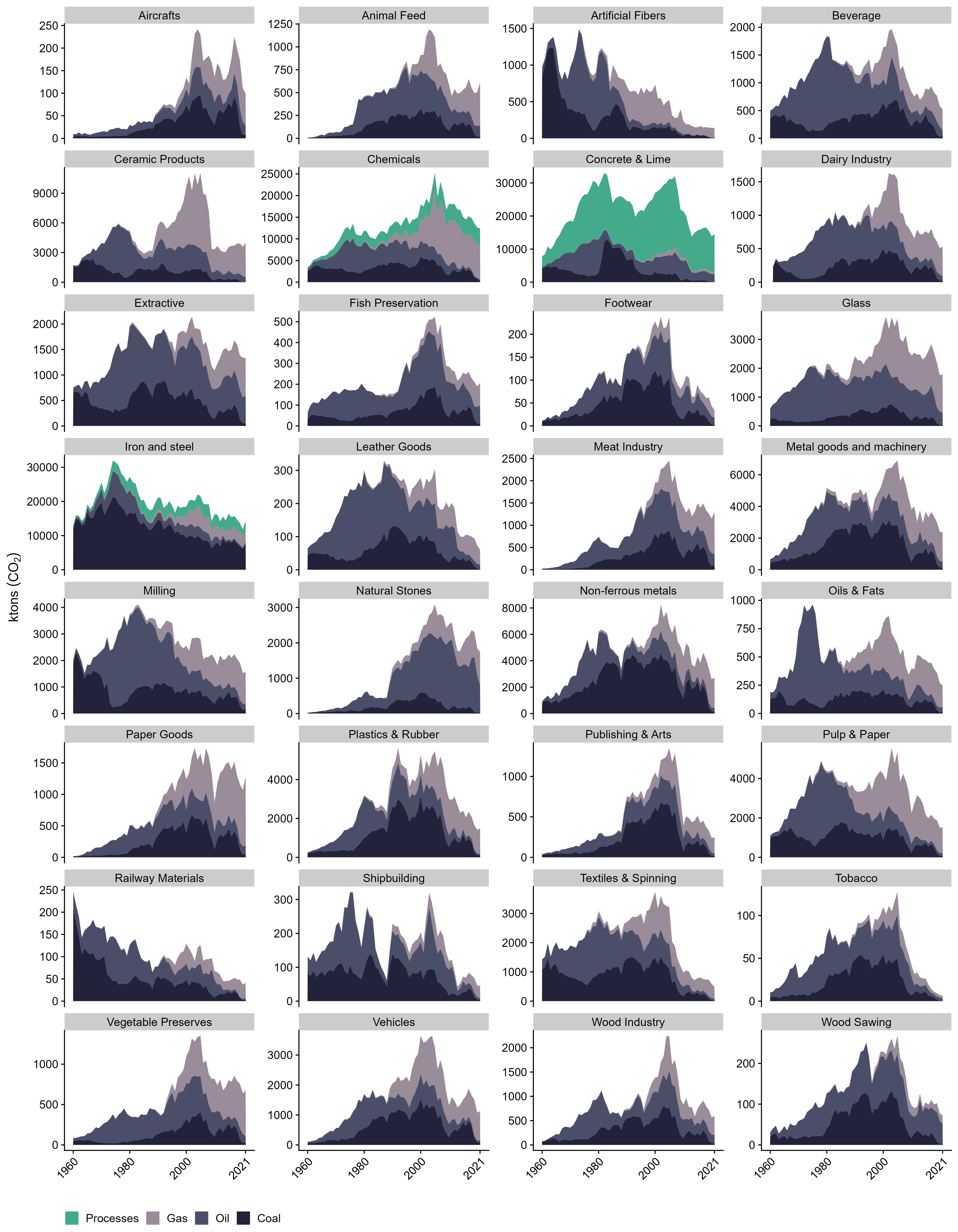


*Fig. 10 CO_2_ emissions in industrial sub-sectors by emission source*


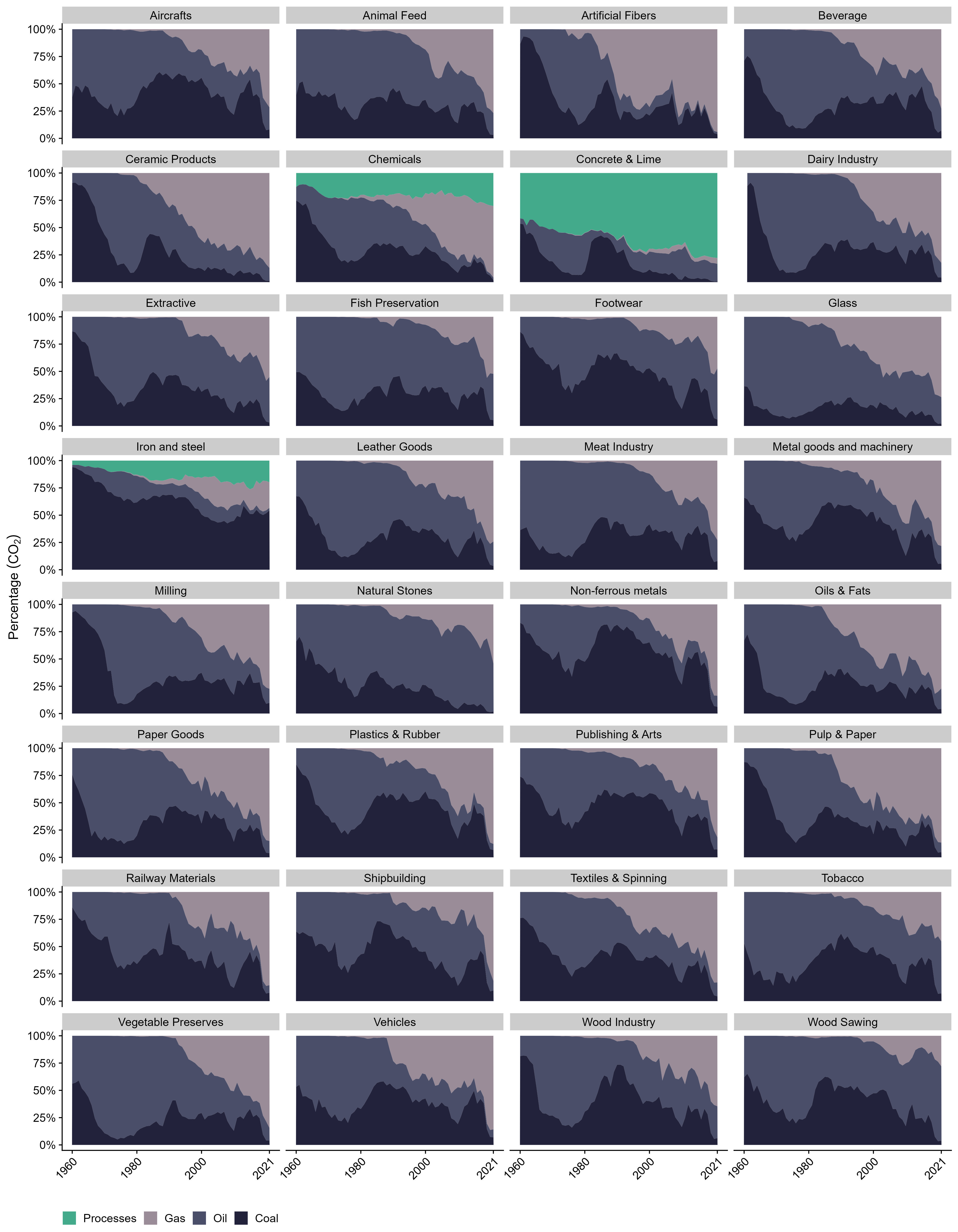


*Fig. 11 Percentage distribution of CO_2_ emission sources within each industrial sub-sector*

- 1. **Sensitivity analysis**

Given the wide variety of historical sources, the harmonization decisions, and the estimation methods adopted for certain sectors in reconstructing the series presented in this paper, a sensitivity analysis was carried out to assess the extent to which these choices affect the results and the robustness of the estimates. Alternative bottom-up methods were applied to estimate energy consumption in iron and steelmaking, metallurgy, cement, ceramic products, and chemicals prior to 1950.

One of these alternatives relied on local regression (LOESS) under the assumption of uniform annual efficiency across all blast furnaces. In practice, furnace efficiencies varied considerably across regions, depending on production scale and the type of fuel employed. By disregarding these regional differentials, the results may be biased toward the higher efficiencies of the main producing areas, particularly Vizcaya. However, at the national scale the discrepancy is minimal and affects the overall results only marginally (Fig. 12).


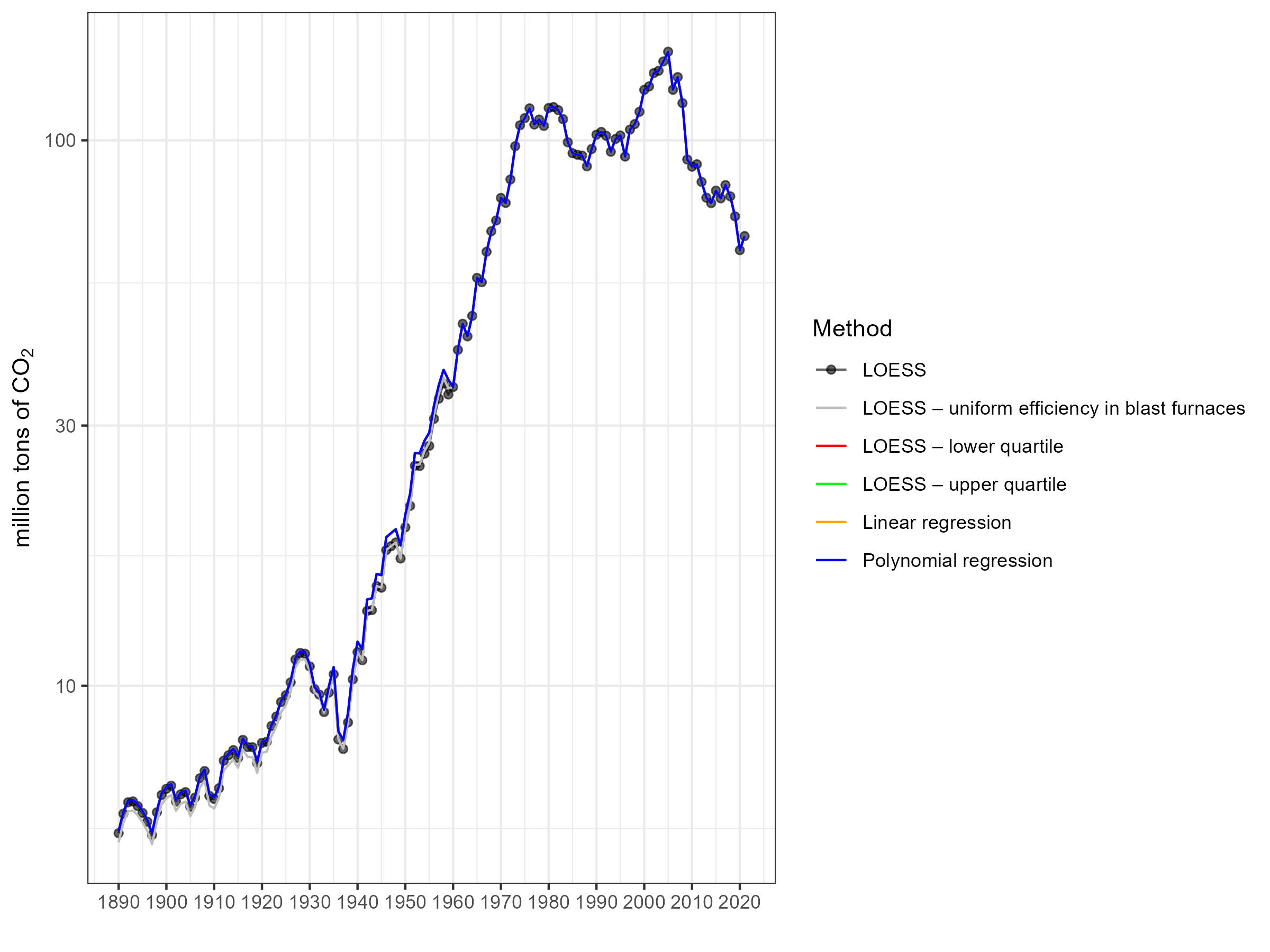


Fig. 12 Comparison of Estimation Methods for the Sensitivity Analysis of Total Industrial CO₂ Emissions (Mtons)

A second test applied LOESS using the lower quartile of efficiency values as the reference. While this does not affect aggregate results—since it merely reshapes the distribution of total emissions—it yields substantial sectoral divergences (Figs. 13 and 14). In 1925, emissions appear 28% lower in iron and steel, 58% lower in concrete and lime, but 103% higher in Consumer Goods and 326% higher in Capital Equipment. This pronounced error margin reflects two factors: first, the strong variability in efficiency across heavy industries, shaped by regional asymmetries in resource quality and access to foreign technologies, which were particularly marked during Spanish uneven first industrialization; and second, the sensitivity of Capital Equipment and Consumer Goods to residual calculations, as their estimates depend on the differential once heavy-industry consumption is accounted for.

The inverse bias emerges when LOESS is applied using the upper quartile of efficiency values, that is, when the least efficient plants are assumed to set the sectoral average. In this case, emissions in iron, steel, and cement are overestimated, while those in Capital Equipment and Consumer Goods are underestimated.

The preference for local regression is justified by the need to capture abrupt and asymmetric shifts in efficiency over time. To test whether this choice influences the results, we also estimated the series using standard linear and polynomial regressions. These alternatives again left aggregate emissions virtually unchanged but introduced some sectoral discrepancies. In 1925, deviations reached 2.1% in iron and steel, 4% in cement and concrete, 50% in Capital Equipment, and 9% in Consumer Goods. Sectoral shares of total emissions vary less, with Capital Equipment shifting by only 1.8 percentage points. This confirms that, compared to linear regression, local regression is crucial for identifying significant temporal discontinuities in efficiency and for allocating CO₂ emissions accurately, while exerting little influence on the aggregate sectoral distribution.


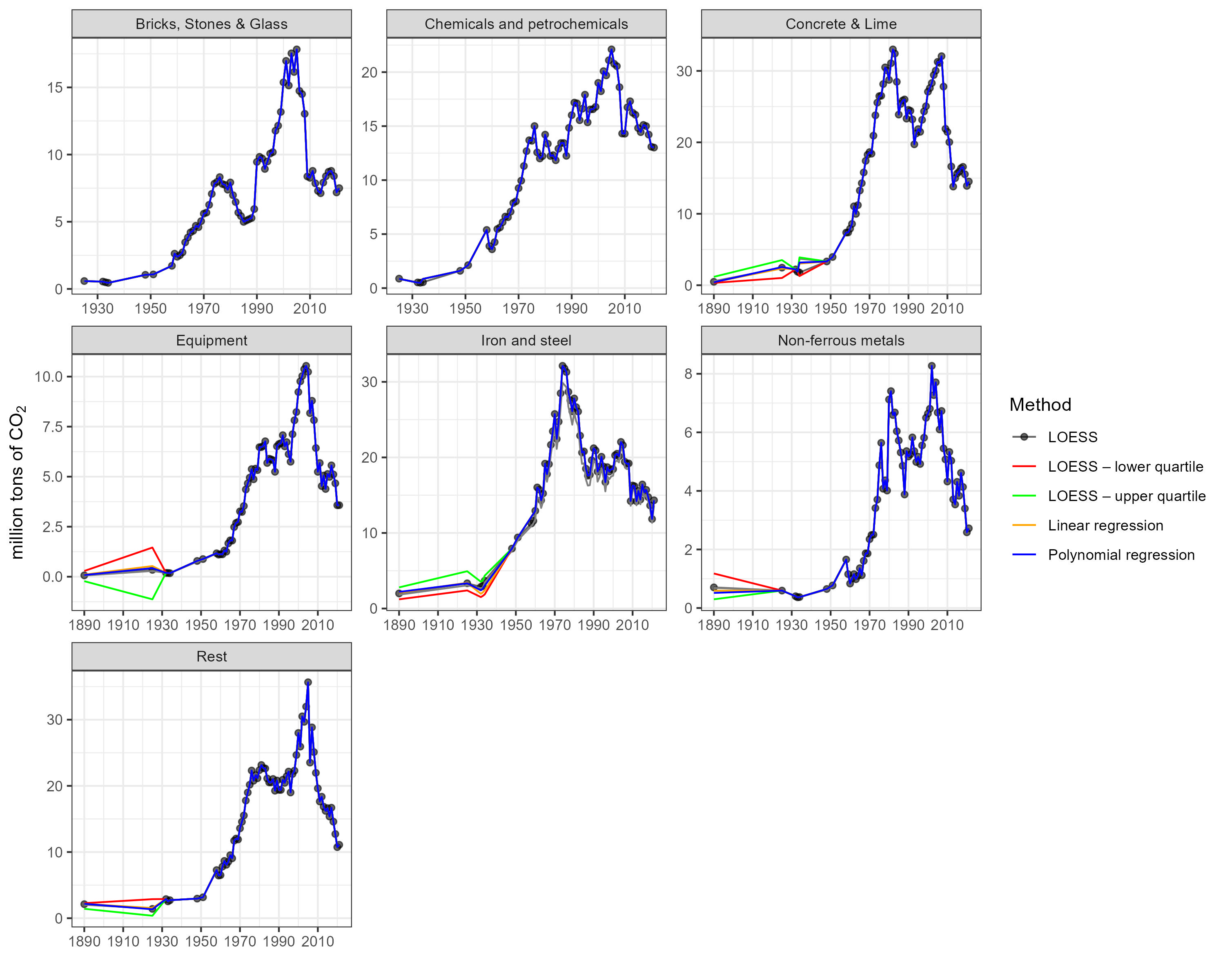


*Fig. 13 Comparison of Estimation Methods for the Sensitivity Analysis of Sectoral Industrial CO₂ Emissions (Mtons)*


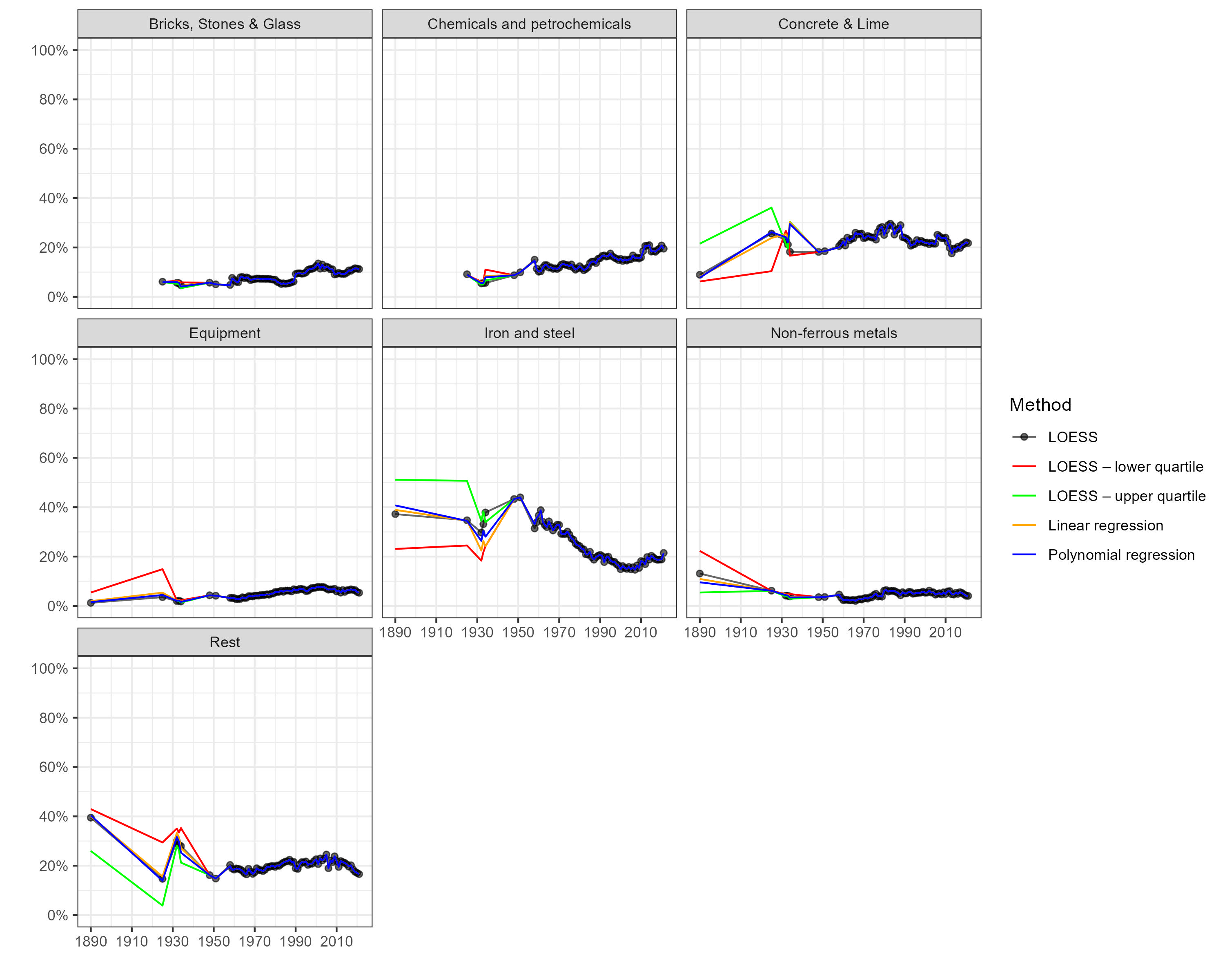


*Fig. 14 Comparison of Estimation Methods for the Sensitivity Analysis of Subsectoral Industrial CO₂ Emissions (%)*

Overall, the sectors most sensitive to parameter variation are Capital Equipment and Consumer Goods. We conclude that local regression is the most appropriate method, as it captures the abrupt shifts in productivity associated with technological imports and protectionist policies between 1890 and 1934. The comparison with linear regression results underscores that non-linearity is fundamental to the model.

1. **Decomposition and decoupling analysis**
   1. **Decomposition analysis: variables and periodization**

**We analyze the drivers of CO_2_ emissions (C) for the entire industry and for each sector and sub-sector, using a Logarithmic Median Divisia Index (LMDI) additive decomposition analysis (Ang, 2005). In our model, variations in C are explained by changes in activity volume (A), represented by value added (€2010), and by changes in the energy intensity of value added and the carbon intensity of energy consumption. The latter is defined as CO_2_ emissions per GJ of final energy consumed (m), and is primarily determined by the energy mix. Energy intensity of value added, on the other hand, is defined as GJ of final energy consumed per € of value added (e), and is determined by technological changes and production structure (see Table 2). Finally, structural change captures the share of each industrial subsector in total value added (Aᵢ/A).**

| **Variable** | **Description** | **Changes** |
| --- | --- | --- |
| C | CO_2_ emissions | Variation in CO_2_ emissions |
| A | Activity: measured as Value Added in 2010 euros | Value Added is a proxy for economic activity. It is estimated as gross income from operating activities minus intermediate costs and taxes. Its variations depend on the level of production and costs |
| E | Energy intensity of production: final energy per € of value added | Its improvement can be explained by technological changes, for example, more efficient machinery, and by changes in the production structure, that is, by a greater growth in cleaner sectors |
| M | Carbon intensity of energy use: mainly determined by the energy mix, that is, the share of each energy source in total energy consumption | Its variation is due to changes in the types of energy consumed, as the emissions per unit of energy differ depending on the type of energy. |
| S | Structural change: share of each subsector in total value added in industry | This component reflects the impact of shifts in the relative weight of different branches on total emissions |

*Table 6. Variables and drivers of CO₂ emissions changes used in the decomposition analysis.*

The choice of periodization in a decomposition analysis is of critical importance, as the results are highly sensitive to the specific benchmarks selected. Different periodization can lead to substantially different interpretations of structural change, growth dynamics, or emission trends. For the pre-1958 analysis, the choice of periods is determined by the availability of statistical information. As no annual sectoral data exists before that date, only benchmark years marking milestones in industrial development are available. As a result, the intervals are inevitably of unequal length—for example, 35 years between 1890 and 1925, 9 years between 1925 and 1934, and 17 years between 1934 and 1951.

Although such asymmetry may appear to be a methodological limitation, it reflects the constraints of historical sources and aligns with standard practice in economic historiography, which relies on benchmarks when annual series are unavailable. In this sense, comparisons between long and short intervals before 1951 are not directly equivalent to the subsequent analysis, but they allow us to reconstruct the main long-term trends and connect them to the period for which annual data exist (from 1958 onward).

The decomposition analysis presented in the paper is based on the periodization proposed by Carreras and Tafunell (2018) from 1959 onward, which is widely used in Spanish economic and industrial historiography. This choice situates the results within a recognized historical narrative and ensures comparability with other studies. However, to assess the reliability of this approach, we contrasted the results with additional procedures: a structural breaks analysis and a five-year moving average scheme.

The first exercise consisted of identifying statistically significant structural changes. A structural breaks analysis was performed to detect potential breakpoints in the emissions time series (1890–2021), the variable was first transformed into an annual series and tested for stationarity using the Augmented Dickey–Fuller (ADF) test. Optimal breakpoints in the relationship between emissions and time were then estimated by comparing the Bayesian Information Criterion (BIC) with the residual sum of squares. Confidence intervals at the 95% level were calculated to locate the breaks more precisely, which were then plotted alongside the evolution of the series.


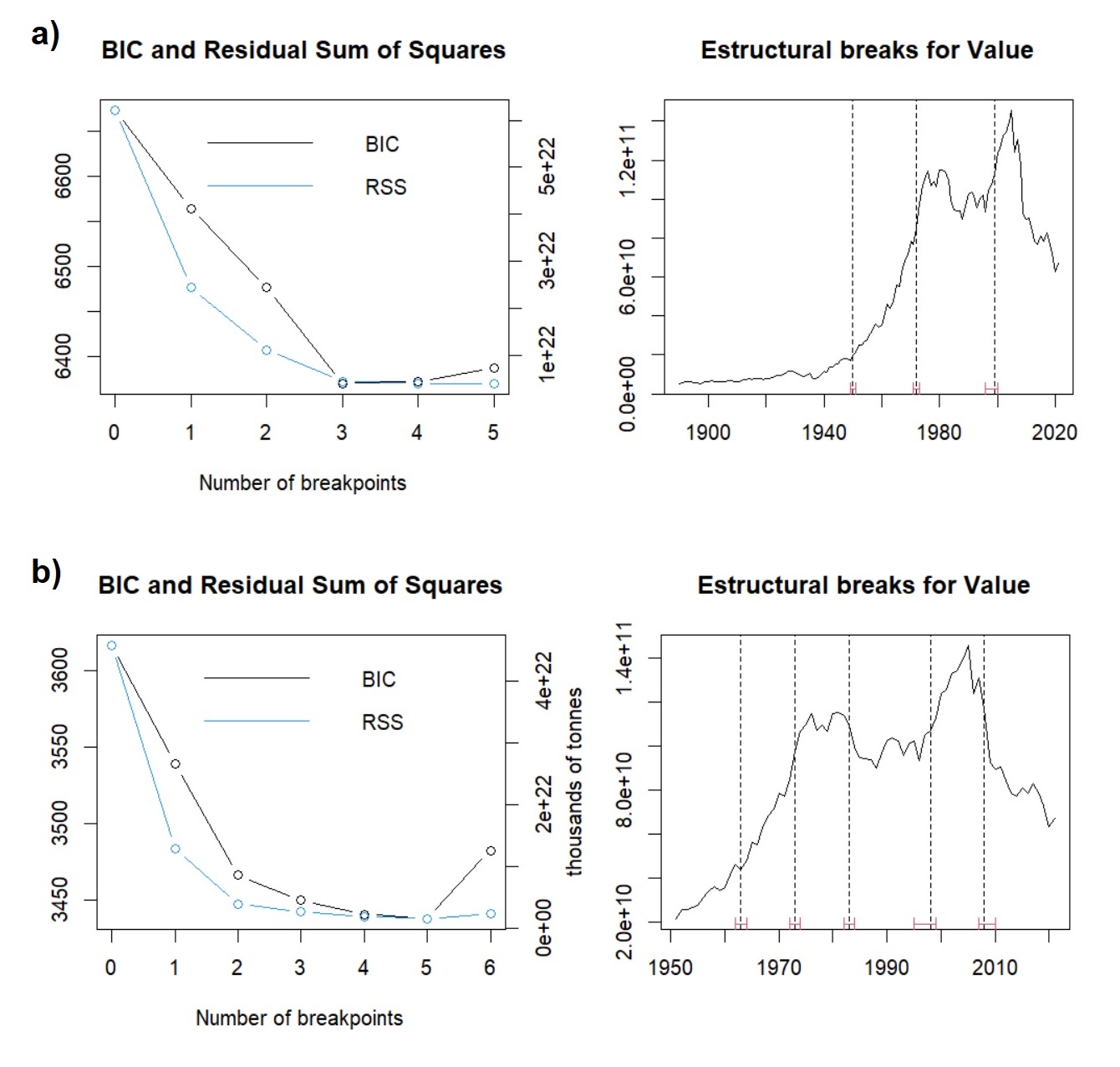


*Fig. 15 Structural breaks (a) in the full series (1890-2021) and (b) in the series 1951-2021*

Applied to the full 1890–2021 series, the analysis identified three main breaks in 1950, 1972, and 1999 (see Fig. 15). However, because the largest shifts occur after 1950 and no sectoral information is available for that year, the procedure was repeated for the period 1951–2021. In this case, the identified breakpoints were in 1963, 1973, 1983, 1998, and 2008.

The decomposition results obtained using this periodization do not fundamentally differ from those presented in the paper. As shown in Fig. 16a, output growth was the main driver during the second decade of the twentieth century. The decline in emissions between 1999 and 2021 is explained by improvements in both energy and carbon intensity, with production playing only a minor role, since output varied only moderately between those two points in time. A limitation of this periodization, however, is that it overlooks key tipping points that occurred within this timeframe—most notably, the acceleration of economic activity up to 2007 and the onset of the Great Recession thereafter.


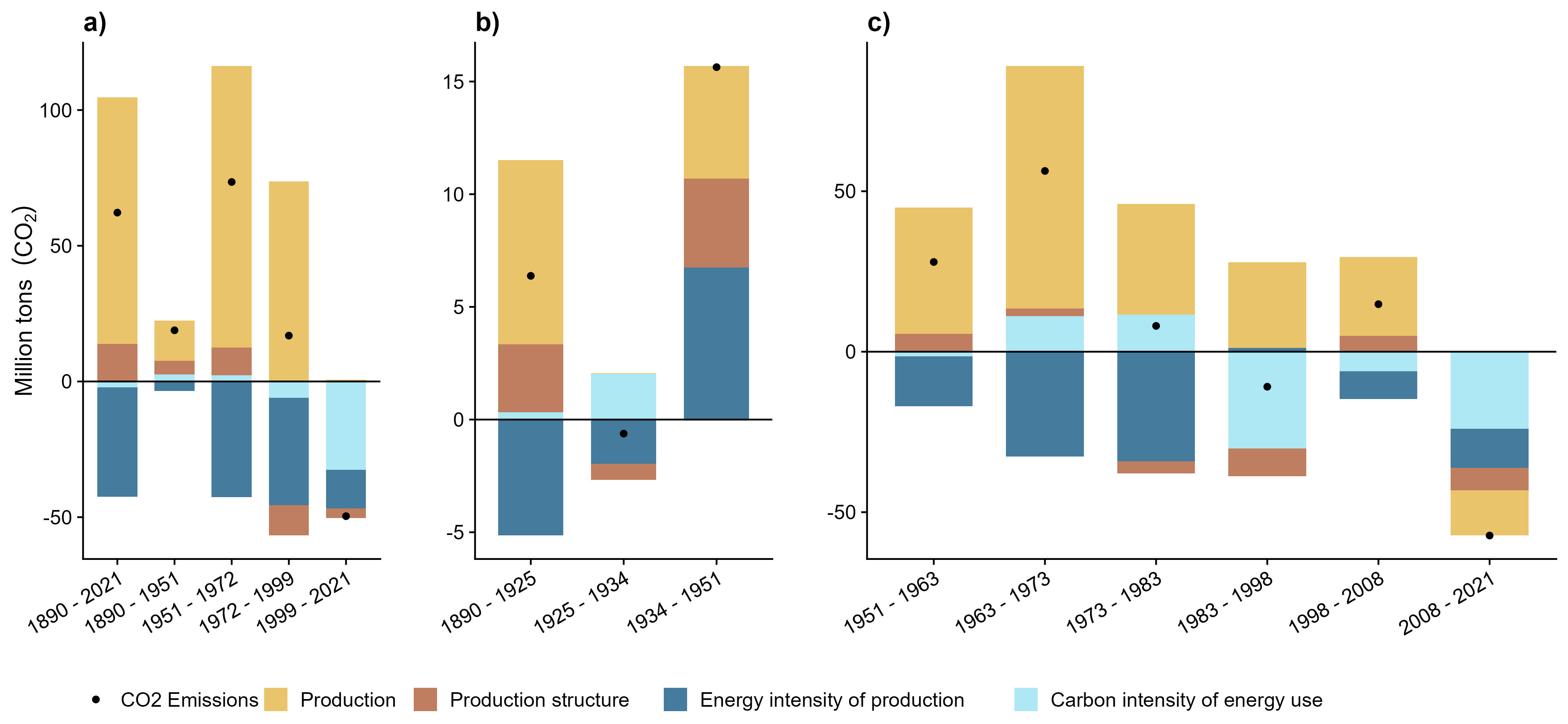


*Fig. 16 Decomposition Analysis with Periodization Based on Structural Break Analysis in the full series (a), in the series 1890-1951 (b) and in the series 1951-2021 (c)*

As these major structural changes must be acknowledged, Fig. 16c applies the periodization derived from the structural breaks analysis for 1951–2021, and reflects a very similar pattern to the decomposition presented in the paper. In this case, energy intensity appears even more clearly as the main counterforce to emissions up to the 1980s, while carbon intensity becomes the dominant factor thereafter. The latter shows an increase between 1963 and 1983, reflecting two dynamics not fully captured in the decomposition analysis of the paper: the substitution of coal with hydropower in electricity generation, and the replacement of coal with oil in cement production. As a result, the decline in carbon intensity from 1983 onward is even more pronounced under this alternative periodization. From 1998, however, the results are virtually identical to those reported in the paper.

Second, we employed five-year schemes to capture local variations not reflected in the results of the paper. Both simple five-year averages and five-year moving averages were applied to smooth annual volatility and explore shorter-term fluctuations. The two approaches (Figs. 17 and 18) offer trends very similar to those previously discussed, but they also reveal that during the first half of the 1980s emissions were not driven by output growth, due to the industrial crisis—a pattern obscured in longer-period aggregations.


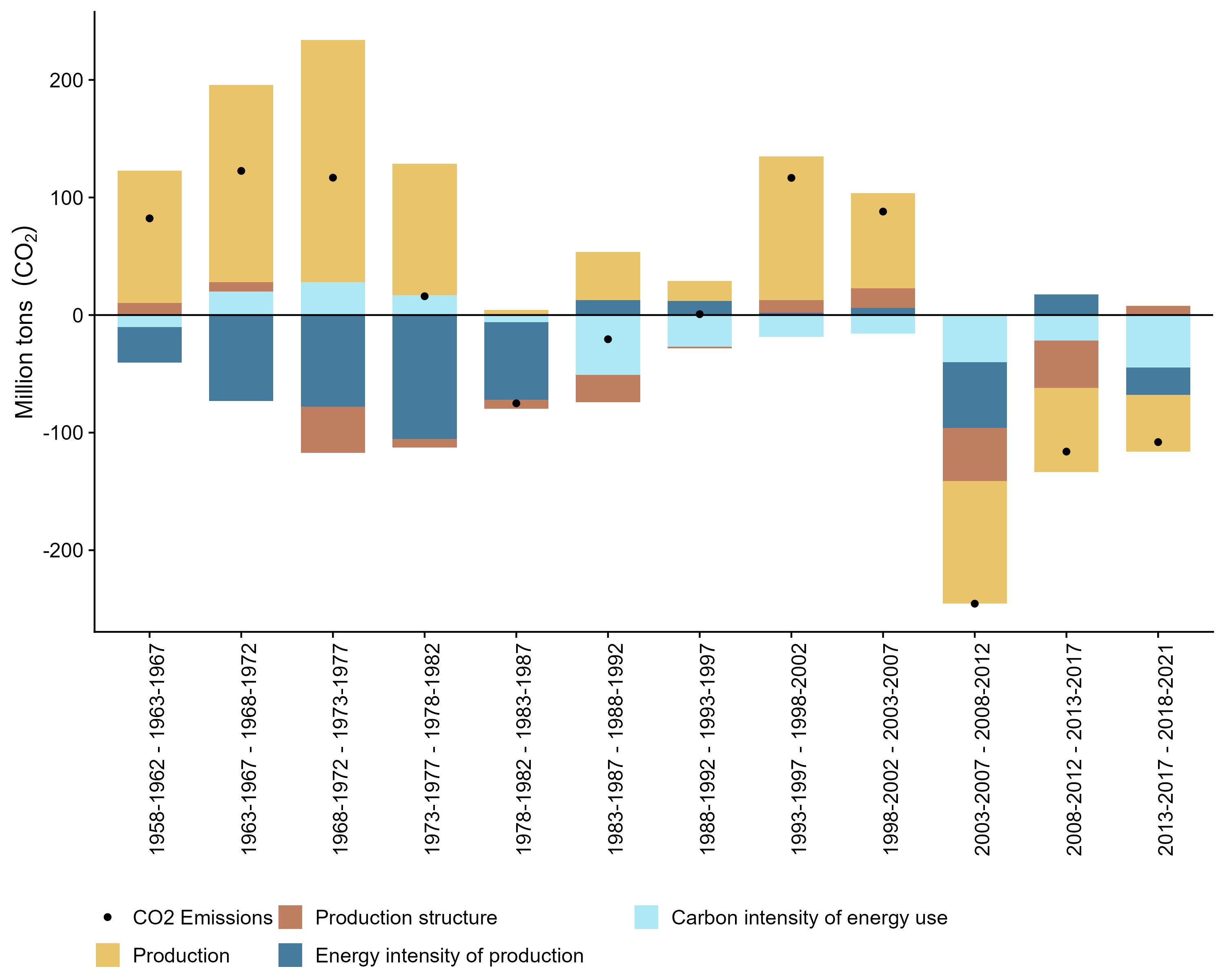


*Fig. 17 Decomposition Analysis with Periodization Based on Five-Year Simple Averages*

The decomposition analysis based on five-year moving averages (Fig. 17) further highlights the role of short-term changes in the drivers. Specifically, improvements in energy intensity are shown to exert a more sustained effect at certain points—such as the late 1970s, the early 1980s, and the post-2007 period—while variations in the carbon intensity of energy use appear more gradual, with a comparable impact in the short run.


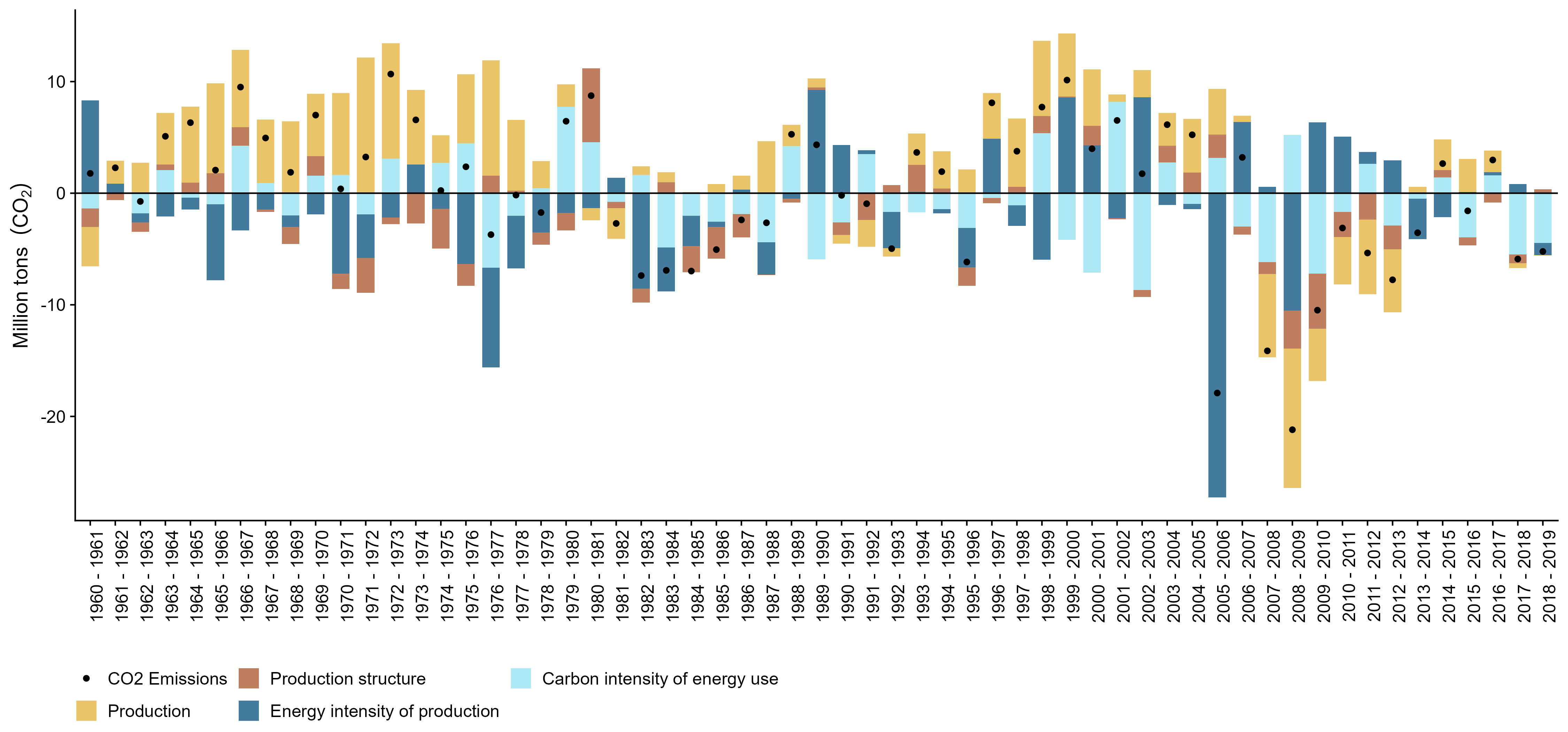


*Fig. 18 Decomposition Analysis Using a Five-Year Moving Average Periodization*

These tests show that, although the results of the decomposition analyses are sensitive to the choice of periodization, the core conclusions remain consistent. Alternative periodization provides complementary insights that both explain and reinforce the main arguments advanced in the paper.

- 1. **Decoupling analysis: scenarios and periodization**

In this study, we assess the degree of decoupling between value added and CO₂ emissions using a model inspired by Tapio (2005). This approach compares the growth rates of emissions and value added and classifies their joint evolution into a set of scenarios, five of which are presented in Table 7.

| **Scenario** | **Value Added (2010€)** | **CO₂ emissions (kg)** | **Decoupling** | **Interpretation** |
| --- | --- | --- | --- | --- |
| Dirty Growth | Increase slower than CO₂ emissions | Increase faster than value added | No | Environmentally inefficient industrial growth |
| Weak Decoupling | Increase faster than CO₂ emissions | Increase slower than value added | Yes | Industrial growth with relative environmental efficiency gains, but without reducing total environmental impact |
| Strong Decoupling | Increase | Decrease | Yes | Industrial growth accompanied by absolute reductions in environmental impacts |
| Recessive Mitigation | Decrease | Decrease | Yes | Industrial recession with declining environmental impacts (e.g., downturns in which the most polluting industries contract more sharply) |
| Dirty Recession | Decrease | Increase | No | Industrial recession with rising environmental impacts (e.g., crises in which ageing equipment increases inefficiency) |

*Table 7. Scenarios and Variables used in the Decoupling Analysis*

Because the carbon intensity of production can improve either through reductions in energy intensity or through a cleaner energy mix, we conduct an additional exercise to determine which mitigation strategy has played the more significant role. We distinguish four scenarios, defined in Table 8.

| **Scenario** | **Energy intensity (GJ/2010€)** | **Carbon intensity of energy (kgCO₂/GJ)** | **Interpretation** |
| --- | --- | --- | --- |
| Energy Mix Efficiency | Increase | Decrease | Adoption of lower-carbon energy sources without energy savings (e.g., decarbonizing the electricity mix while maintaining an inefficient industrial structure) |
| Energy Intensity Efficiency | Decrease | Increase | Cost-saving strategies combined with greater reliance on carbon-intensive fuels (e.g., a more carbon-intensive electricity system during industrial electrification) |
| Dual Efficiency | Decrease | Decrease | Simultaneous improvements in both energy intensity and the carbon intensity of energy use |
| Dual Inefficiency | Increase | Increase | Deterioration of both energy intensity and the energy mix |

*Table 8. Scenarios used to assess Efficiency Pathways*

As with the decomposition analysis, the results of the decoupling analysis are sensitive to the chosen periodization. We therefore conducted a sensitivity exercise analogous to that described in Section 4.1. Using the same database, we applied (i) the periodization derived from the structural breaks analysis (Fig. 19) and (ii) a simple five-year averaging scheme starting in 1960 (Fig. 20).

The sensitivity analysis shows only minor differences between the structural-break periodization and that used in the main text (Fig. 19a). The structural-break scheme highlights several episodes of dirty growth between 1951 and 1963 and, more notably, places the period of strong decoupling between 1983 and 1998, whereas the main text situates it between 1973 and 1986. This discrepancy arises because the strongest relative decoupling occurs between 1983 and 1986, and the choice of benchmark allocates this effect to one interval or the other. Using the 1973–1986 interval is analytically preferable, as industrial output resumed growth around 1986 after several years of restructuring policies.


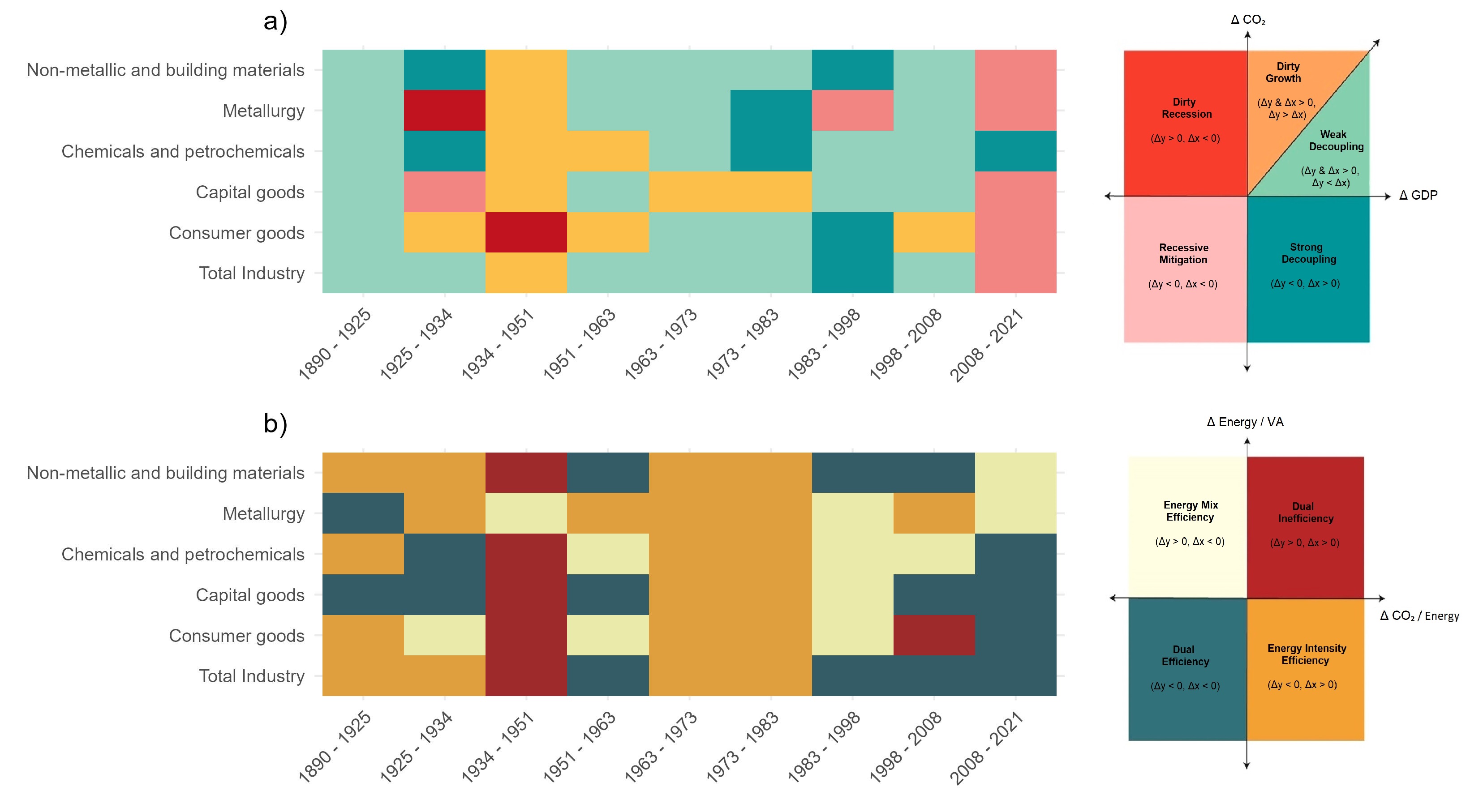


Fig. 19 Comparative evolution of value-added growth and CO2 emissions (a); and energy intensity of value added versus carbon intensity of energy use (b), using structural breaks periodization (see section 4.1)

In the efficiency analysis, the trends identified in the paper—namely, the predominance of energy-intensity improvements up to the 1980s and of reductions in the carbon intensity of energy use thereafter—are, if anything, reinforced. Nonetheless, two differences merit discussion. Under the structural-break periodization (Fig. 19b), the period 1973–1983 corresponds to an energy intensity efficiency scenario, whereas in the 1973–1986 interval used in the main text it is classified as dual efficiency. This reflects a temporary return to coal in electricity generation during the early 1980s (Muñoz & Rubio, 2024), subsequently reversed (see Fig. 9). This short-lived reversal is not captured when aggregating the broader 1973–1986 period, but its effects were local and did not alter long-term dynamics.

A second difference concerns the period 1998–2008, classified as dual efficiency in the structural-break scheme but as energy mix efficiency in the main text. This occurs because Fig. 19 captures the effects of the 2007 crisis within the 1998–2008 range; the downturn hit the most energy-intensive sectors (e.g., cement production) particularly hard, artificially suggesting a decline in energy intensity across the entire period. As shown in the main text, this was not the case.

We also conducted a decoupling analysis based on five-year simple averages from 1960 onward (Fig. 20). Although the main trends remain consistent with those in the paper, several differences appear, some of which coincide with and are already explained in Fig. 19—such as the energy mix efficiency episode between 1980 and 1985. In this same interval, Fig. 20a shows a recessive mitigation scenario, because the five-year window captures the early-1980s recession, whereas the main text begins from a lower value-added level in 1973, before output had reached its peak. Five-year averages also allow identification of short-term dirty growth episodes across sectors in the early 1960s, at the onset of the developmentalist phase, and in the early 2000s during the housing boom. However, these episodes are short-lived, and their relevance for a long-term analysis such as ours is limited.


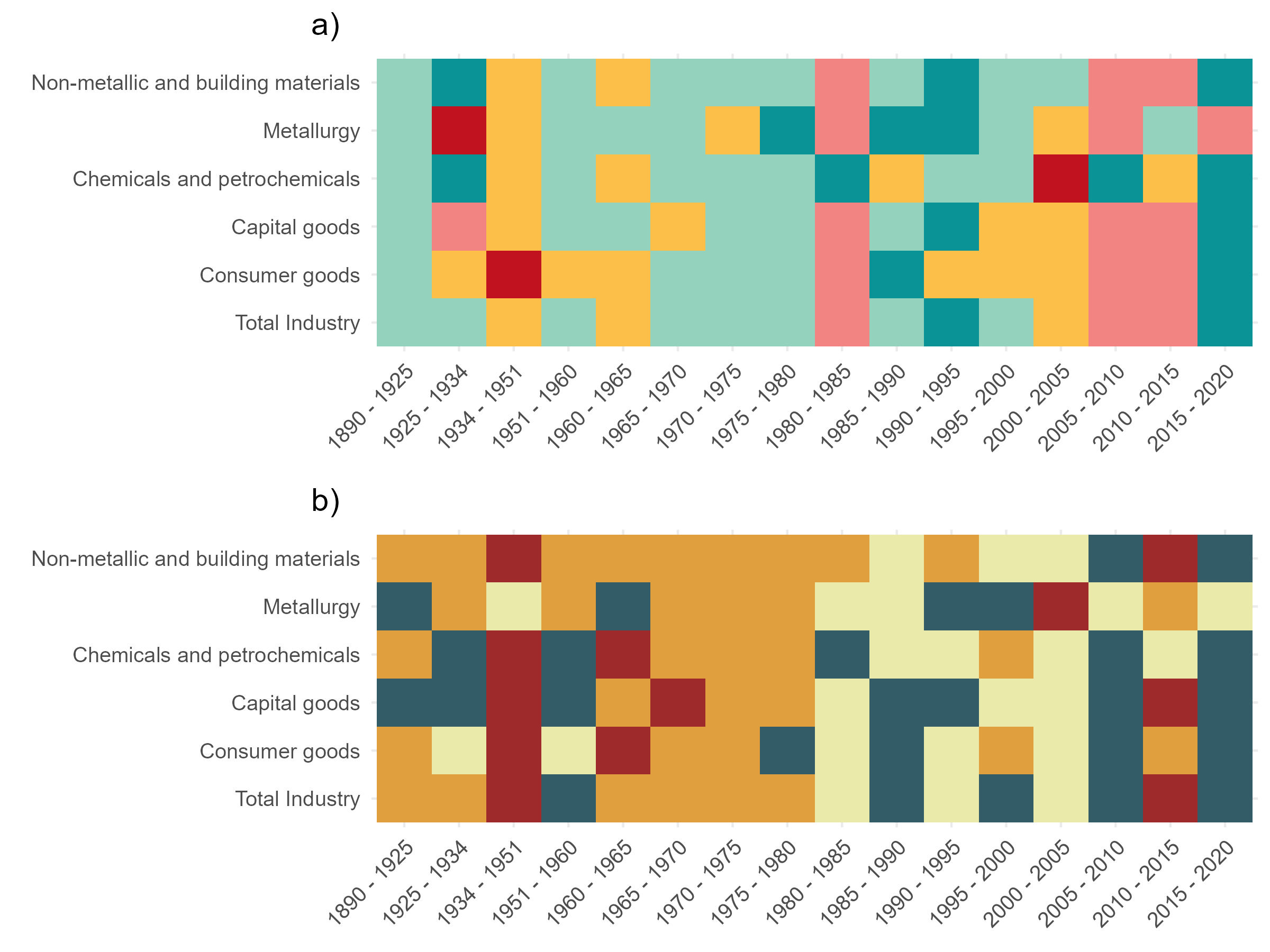


Fig. 20 Comparative evolution of value-added growth and CO₂ emissions (a); and energy intensity versus carbon intensity (b), using periodization based on five-year simple averages

1. **International database**
   1. **International sources and data**

No long-term series of industrial CO₂ emissions exist for most countries prior to 1970, which limits the possibility of comparing Spain’s historical trends with those of other economies. To shed light on whether late industrialisation is associated with structurally carbon-intensive trajectories, this study reconstructs carbon-intensity series for several late-industrialising Southern European economies (Portugal, Italy, Greece) and for a group of first-comers or advanced industrial economies with higher value-added manufacturing structures (the United Kingdom, Germany, France, and Sweden).

The dataset offering the longest temporal coverage is the EDGAR Community GHG Emissions Database (Crippa et al., 2023), which reports direct CO₂ emissions from manufacturing aggregated with the construction sector, as well as process-related CO₂ emissions from cement, metals, chemicals, glass, and lime for the period 1970-2023. However, EDGAR does not provide emissions disaggregated by manufacturing subsector, nor does it include indirect CO₂ emissions arising from electricity consumption, since “Main Activity Electricity and Heat Production” is treated as a separate sector without allocating produced electricity to final users.

Eurostat’s energy balances offer a homogeneous and internationally comparable framework for analysing the evolution of European energy systems between 1990 and 2023. For the electricity sector, these balances provide detailed information on the contribution of each primary and secondary energy source to electricity generation. They report the inputs to transformation plants (primary production, imports and exports, stock changes, and transfers), together with the energy consumed within the transformation process itself. Based on these inputs, the balances present the outputs of electricity and heat generation, enabling the assessment of generation efficiency and technological change. The balances also include own use of energy by the energy industry and network distribution losses, allowing the calculation of electricity effectively available for final consumption.

Regarding final energy use, Eurostat supplies disaggregated consumption data for twelve standardised economic sectors, including industry, transport, households, services, agriculture, construction, fishing, non-energy use, and other country-specific sectors. For each sector, the balances detail final consumption across a wide range of more than twenty energy sources, including fossil fuels, electricity, heat, biomass, waste, and other renewables. This sector–source structure yields a comprehensive set of internationally comparable final energy consumption matrices, suitable for analysing demand patterns, energy substitution processes, and sectoral profiles of energy intensity.

The Eurostat database covers all EU Member States, EFTA countries, and additional European countries included in harmonised energy statistics. Its annual series for 1990–2023 ensures methodological consistency, enabling the study of energy transitions, the estimation of energy-related CO₂ emissions, and the historical reconstruction of sectoral and source-specific energy use patterns.

To compute carbon intensity, value-added data at both current prices and constant 2015 prices were sourced from the United Nations Statistics Division (UNSD). The dataset covers 267 countries and regions, although temporal coverage varies across cases; for example, manufacturing value-added data for Greece and Portugal are only available from 1995 onwards.

- 1. **Estimation**

Using Eurostat energy balances, it is possible to estimate primary energy consumption in manufacturing in a manner consistent with Aguilera et al. (2019) and to replicate the methodology employed in this article—described in Section 3—to estimate both direct and indirect CO₂ emissions by country and manufacturing sector from 1990 onwards. The procedure involved four steps applied to electricity consumption data to determine the contribution of primary energy sources at the national level.

First, the composition of electricity generation by energy source was calculated for each country using Eurostat’s electricity generation data (Eurobase code GEP).

Second, the average efficiency of the electricity generation sector was estimated by comparing total primary energy inputs to electricity generation (Eurobase code TI_EHG_E), excluding energy used internally by the energy sector (code NRG_EHG_E), with total electricity output (GEP). This comparison yielded an implicit efficiency coefficient for electricity generation in each country.

Third, transmission and distribution losses were estimated by comparing gross electricity production (GEP) with electricity available for final consumption (Eurobase code AFC). These losses were used to adjust the primary energy input requirements, producing a more accurate estimate of the primary energy effectively consumed to supply electricity to end users.

Primary energy data were then converted into direct and indirect CO₂ emissions using the coefficients reported in Table 5. Process-related CO₂ emissions were taken directly from the EDGAR Community GHG Emissions Database (Crippa et al., 2023).

Carbon intensity was calculated by dividing manufacturing CO₂ emissions by manufacturing value added, expressed in constant 2015 prices. Value-added data were obtained from the United Nations Statistics Division (UNSD, 2025).

**REFERENCES**

Dirección General de Agricultura, Industria y Comercio (various years) *Estadística Mineral y Metalúrgica de España*, Madrid, 1861 to 1957.

Eurostat, 2024. *European Energy Statistics Glossary*.

IDAE, 2024. *Balance energético de España: Serie histórica 1990-2021*.

INE, 1951. *Anuario Estadístico de España*, Madrid.

INE, various years. *Encuesta de Consumos Energéticos*, Madrid, 2001 to 2021

INE, various years. *Encuesta Industrial de Empresas*, 1993 to 2013

INE, various years. *Encuesta Industrial*. Madrid, 1978 to 1992.

INE, various years. *Estadística estructural de empresas: sector industrial*, 2014 to 2021

INE, various years. *Estadística Industrial de Energía Eléctrica*, Madrid, 1958 to 1989.

INE, various years. *Estadística Industrial* *de España*. Madrid, 1958 to 1977.

Aguilera, E., Vila-Traver, J., Deemer, B. R., Infante-Amate, J., Guzmán, G. I., & González de Molina, M. (2019). Methane Emissions from Artificial Waterbodies Dominate the Carbon Footprint of Irrigation: A Study of Transitions in the Food–Energy–Water–Climate Nexus (Spain, 1900–2014). *Environmental science & technology*, 53(9), 5091-5101. https://doi.org/10.1021/acs.est.9b00177

Anes Álvarez, R. (1988). La industrialización de Asturias en el siglo XIX: una transformación económica parcial, in *La industrialización del norte de España*, Crítica, 99-112.

Anes, R., & Ojeda, G. (1983). La industria asturiana en la segunda mitad del siglo XIX: de la industrializacion a la expansion hullera, *Revista De Historia Económica / Journal of Iberian and Latin American Economic History*, 1(2), 13-29. <https://doi.org/10.1017/S0212610900012659>

Ang, B. W. (2005). The LMDI approach to decomposition analysis: A practical guide. *Energy policy*, 33(7), 867-871. https://doi.org/10.1016/j.enpol.2003.10.010

Bartolomé, I. (2007). La industria eléctrica en España (1890-1936). Banco de España.

Bilbao, L. M. (1984). La siderurgia vasca, 1720-1885 atraso tecnológico: Política arancelaria y eficiencia económica. En *Antecedentes próximos de la sociedad vasca actual, siglos XVIII y XIX*, Sociedad de Estudios Vascos = Eusko Ikaskuntza, 79-93.

Bilbao, L. M. (1985). Renovación tecnológica y estructura del sector siderúrgico en el País Vasco durante la primera etapa de la industrialización (1849-1880). Aproximación comparativa con la industria algodonera de Cataluña. En *Industrialización y nacionalismo, análisis comparativos: Actas del I Coloquio Vasco-Catalán de Historia, celebrado en Sitges, 20-22 de diciembre de 1982,* Servicio de Publicaciones de la Universidad Autónoma de Barcelona, 211-228.

Bilbao, L. M., & Fernández de Pinedo, E. (1988). Artesanía e Industria. En *Enciclopedia de Historia de España*, Alianza Editorial, 105-190.

Carreras, A. (2005). Industria, *Estadísticas históricas de España: Siglos XIX-XX*, Fundación BBVA, 357-453.

Carreras, A., & Tafunell, X. (2018). *Entre el imperio y la globalización: Historia económica de la España contemporánea*. Critica.

Coll, S., & Sudrià, C. (1987). *El carbón en España, 1770-1961*. Turner.

Corbera Millán, M. (2001). *La Siderurgia Tradicional en Cantabria*. Septem Ediciones, S.L.

Crippa, M., Guizzardi, D., Schaaf, E., Monforti-Ferrario, F., Quadrelli, R., Risquez Martin, A., Rossi, S., Vignati, E., Muntean, M., Brandao De Melo, J., Oom, D., Pagani, F., Banja, M., Taghavi-Moharamli, P., Köykkä, J., Grassi, G., Branco, A., & San-Miguel, J. (2023). *GHG emissions of all world countries: 2023*. 31658.

Errandonea, E. (1935). Interconexión eléctrica. *Revista de Obras Públicas*, 83(2683), 457-464.

Escudero, A. (2005). La hegemonía siderúrgica de Vizcaya: Un análisis de costes (l890-1913). *Revista de historia industrial*, 28, 47-79.

Escudero, A., & Parejo, A. (2015). Malaga Steel Industry (1899-1924). *Revista de historia industrial*, 58, 319-347.

Eurostat. (2019). *Energy balance guide. Methodology guide for the construction of energy balances & Operational guide for the energy balance builder tool*. European Comission.

Fernández de Pinedo, E. (1985). La industria siderúrgica, la minería y la flota vizcaína a fines del siglo XIX. Unas puntualizaciones. En *Mineros, sindicalismo y política*, Fundación José Barreiro, 149-177.

Fernández de Pinedo, E. (1988). Factores técnicos y económicos en el origen y desarrollo de la moderna siderurgia y la flota vizcaína, 1880-1899. En *La industrialización del Norte de España*, Crítica, 252-280.

Fouquet, R. (2008). *Heat, Power and Light. Revolutions in Energy Services*. Edward Elgar.

Gales, B., Kander, A., Malanima, P., & Rubio-Varas, M. (2007). North versus South: Energy transition and energy intensity in Europe over 200 years. *European review of economic history*, 11(2), 219-253. https://doi.org/10.1017/S1361491607001967

García, S., Francisco José Goerlich Gisbert, & Vicente Orts Ríos. (1994). *Macromagnitudes básicas a nivel sectorial de la industria española: Series históricas*. IDEAS Working Paper Series from RePEc.

González Portilla, M. (1985). *La siderurgia vasca: (1880-1901): Nuevas tecnologías, empresarios y Política económica*. Servicio Editorial, Universidad del País Vasco.

Houpt, S. (1998). *Cambio técnico y localización en la siderurgia española integrada* PhD Diss. Universidad Carlos III de Madrid.

Infante-Amate, J., Iriarte-Goñi, I., Urrego-Mesa, A., & Gingrich, S. (2022). From woodfuel to industrial wood: A socio-metabolic reading of the forest transition in Spain (1860–2010). *Ecological Economics*, 201, 107548. <https://doi.org/10.1016/j.ecolecon.2022.107548>

Infante-Amate, J., Travieso, E., & Aguilera, E. (2024). Unsustainable prosperity? Decoupling wellbeing, economic growth, and greenhouse gas emissions over the past 150 years. *World Development*, 184, 106754. <https://doi.org/10.1016/j.worlddev.2024.106754>

Inventario Nacional de Emisiones. (2024). *Tables de datos de reporte (CFR)* [Dataset]. <https://www.miteco.gob.es/es/calidad-y-evaluacion-ambiental/temas/sistema-espanol-de-inventario-sei-.html>

IPCC. (2022). *Mitigation of Climate Change. Working Group III Contribution to the Sixth Assessment Report of the Intergovernmental Panel on Climate Change*. Cambridge University Press.

Iriarte-Goñi, I., & Infante-Amate, J. (2019). Continuity, change, and geographical differences in Spain’s firewood consumption: A new estimation (1860-2010). *Historia agraria*, 77, 33-57. https://doi.org/10.26882/histagrar.077e01i

Kander, A. (2002). *Economic growth, energy consumption and CO2 emissions in Sweden 1800-2000*. PhD Diss. Lund University.

Kander, A., Malanima, P., Warde, P., (2014). *Power to the people: energy in Europe over the last five centuries.* Princeton University Press. <https://doi.org/10.1515/9781400848881>

Lamb, W. F., Wiedmann, T., Pongratz, J., Andrew, R., Crippa, M., Olivier, J. G. J., Wiedenhofer, D., Mattioli, G., Khourdajie, A. A., House, J., Pachauri, S., Figueroa, M., Saheb, Y., Slade, R., Hubacek, K., Sun, L., Ribeiro, S. K., Khennas, S., de la Rue du Can, S., … Minx, J. (2021). A review of trends and drivers of greenhouse gas emissions by sector from 1990 to 2018. *Environmental research letters*, 16(7), 73005. <https://doi.org/10.1088/1748-9326/abee4e>

Liu, N., Ma, Z., & Kang, J. (2015). Changes in carbon intensity in China’s industrial sector: Decomposition and attribution analysis. *Energy policy*, 87, 28-38. <https://doi.org/10.1016/j.enpol.2015.08.035>

Llopis, E., & Fernández, R. (1997). *Índices provinciales y regionales de producción manufacturera, 1964-1977*. Fundación Empresa Pública.

Lucio Villegas, A. (1924). Medios de aumentar el consumo de carbón en España. *Revista Minera*, 75, 298-302.

Malanima, P. (2006). *Energy Consumption in Italia in the 19th and 20th Centuries. A Statistical Outline.* Consiglio Nazionale delle Ricerche. Istituto di Studi sulle Società del Mediterraneo.

Minx, J. C., Lamb, W. F., Andrew, R. M., Canadell, J. G., Crippa, M., Döbbeling, N., Forster, P. M., Guizzardi, D., Olivier, J., Peters, G. P., Pongratz, J., Reisinger, A., Rigby, M., Saunois, M., Smith, S. J., Solazzo, E., & Tian, H. (2021). A comprehensive and synthetic dataset for global, regional, and national greenhouse gas emissions by sector 1970–2018 with an extension to 2019. *Earth system science data*, 13(11), 5213-5252. <https://doi.org/10.5194/essd-13-5213-2021>

Moutinho, V., Varum, C., & Madaleno, M. (2017). How economic growth affects emissions? An investigation of the environmental Kuznets curve in Portuguese and Spanish economic activity sectors. *Energy Policy*, 106, 326-344. <https://doi.org/10.1016/j.enpol.2017.03.069>

Muñoz Delgado, B. and Rubio-Varas, M. del M. (2024) “Transiciones energéticas en España,” in I. Iriarte and J. Infante-Amate (eds.) *Impactos ambientales del crecimiento español contemporáneo. Una perspectiva histórica*. Prensas de la Universidad de Zaragoza, 123–144.

Nadal, J. (1984). Los dos abortos de la Revolución Industrial en Andalucía. En A. Domínguez Ortiz (Ed.), *Historia de Andalucía*, Cupsa, 399-433.

Nadal, J. (2003). *Atlas de la industrialización de España, 1750-2000*. Crítica.

Ojeda, G. (1985). *Asturias en la industrialización española, 1833-1907*. Siglo XXI de España.

Oriol y Vidal, R. (1894). Información oral parlamentaria sobre los Tratados de Comercio presentados en el Senado por el Gobierno de Su Majestad. *Revista Minera*, 2(45), 153-157.

Parejo, A. (2001). Industrialization, desindustrialization and new industrialization in the Spanish regions (1950-2000). *Revista de Historia Industrial — Industrial History Review*, 0(19-20), 15-75. https://doi.org/10.1344/rhi.v0i19-20.19393

Prados de la Escosura, L. (2017). *Spanish Economic Growth, 1850–2015*. Springer Open. <https://doi.org/10.1007/978-3-319-58042-5>

Rissman, J., Bataille, C., Masanet, E., Aden, N., Morrow, W. R., Zhou, N., Elliott, N., Dell, R., Heeren, N., Huckestein, B., Cresko, J., Miller, S. A., Roy, J., Fennell, P., Cremmins, B., Koch Blank, T., Hone, D., Williams, E. D., de la Rue du Can, S., … Helseth, J. (2020). Technologies and policies to decarbonize global industry: Review and assessment of mitigation drivers through 2070. *Applied energy*, 266(C), 114848. https://doi.org/10.1016/j.apenergy.2020.114848

Shan, Y., Zhou, Y., Meng, J., Mi, Z., Liu, J., & Guan, D. (2019). Peak cement‐related CO2 emissions and the changes in drivers in China. *Journal of Industrial Ecology*, 23(4), 959-971. https://doi.org/10.1111/jiec.12839

Shao, S., Yang, L., Gan, C., Cao, J., Geng, Y., & Guan, D. (2016). Using an extended LMDI model to explore techno-economic drivers of energy-related industrial CO2 emission changes: A case study for Shanghai (China). *Renewable & sustainable energy reviews*, 55, 516-536. https://doi.org/10.1016/j.rser.2015.10.081

Urrego-Mesa, A., Infante-Amate, J., & Tello, E. (2025). The shadow of tropical agriculture: Energy transition of Colombian trade-driven agriculture in the 20th century. *Ecological economics*, 231, 108541. https://doi.org/10.1016/j.ecolecon.2025.108541

Zapata, S. (1996). *La industria de una región no industrializada: Extremadura, 1750-1990*. Universidad de Extremadura.
